# Microbial Interactions — Underexplored Links Between Public Health Relevant Bacteria and Protozoa in Coastal Environments

**DOI:** 10.3389/fmicb.2022.877483

**Published:** 2022-06-13

**Authors:** Karolina I. A. Eriksson, Johanna Thelaus, Agneta Andersson, Jon Ahlinder

**Affiliations:** ^1^Department of Ecology and Environmental Sciences, Faculty of Science and Technology, Umeå University, Umeå, Sweden; ^2^Division of CBRN Defence and Security, Swedish Defence Research Agency (FOI), Umeå, Sweden; ^3^Umeå Marine Sciences Centre, Umeå University, Hörnefors, Sweden

**Keywords:** bacteria, protozoa, predation resistance, biotic interactions, aquatic microbiology, co-evolution, joint species distribution model, direct acyclic graph (DAG)

## Abstract

The co-existence of bacteria and protozoa in aquatic environments has led to the evolution of predation defense mechanisms by the bacteria. Some of the predation-resistant bacteria (PRB) are also pathogenic to humans and other mammals. The links between PRB and protozoa in natural aquatic systems are poorly known, but they are important in predicting outbreaks and determining the long-term consequences of a contamination event. To elucidate co-occurrence patterns between PRB (16S rRNA) and bacterivorous protozoa (18S rRNA), we performed a field study in a coastal area in the northern Baltic Sea. Interactions between bacteria and protozoa were explored by using two complementary statistical tools. We found co-occurrence patterns between specific PRB and protozoa, such as *Legionella* and Ciliophora, and we also found that the interactions are genotype-specific as, for example, *Rickettsia*. The PRB sequence diversity was larger in bays and freshwater inlets compared to offshore sites, indicating local adaptions. Considering the PRB diversity in the freshwater in combination with the large spring floods in the area, freshwater influxes should be considered a potential source of PRB in the coastal northern Baltic Sea. These findings are relevant for the knowledge of survival and dispersal of potential pathogens in the environment.

## Introduction

Opportunistic bacterial pathogens often hide in the environment before infecting humans (Matz and Kjelleberg, 2005; Winstanley et al., 2016; Amaro and Martín-González, 2021). They survive in the environment, as they master ecological niches where grazing from protozoa is evaded. Bacteria and protozoa have co-existed in the aquatic ecosystems for a long period, even before the evolution of mammals. As a response to the long-term predation pressure by protozoa, the bacteria have developed defense mechanisms, for example, by changing their size, forming biofilms, excreting toxic compounds, or preventing degradation once engulfed by the protozoa (Matz and Kjelleberg, 2005). Simply described, there are two major forms of predation-resistant bacteria (PRB): those having an extracellular lifestyle (EPRB) and those having an intracellular lifestyle (IPRB) (Weitere et al., 2005; Thelaus et al., 2009).

Some traits that render bacteria resistant to predation also provide tools for infecting mammals, and these are classified as virulence factors (Amaro and Martín-González, 2021). Protozoa, such as ciliates, flagellates, and amoebas, drive the evolution of bacteria as a selective force. Bacterial interactions with protozoa thus play an important role in the development and persistence of virulence traits (Sun et al., 2018). In nature, examples of both potentially pathogenic EPRB and IPRB can be found (Von Reyn et al., 1993; Miyasaka et al., 2006; Kao et al., 2013; Pappa et al., 2017). Despite the importance of associations within the microbial food web, little has been documented regarding PRB–protozoa interactions in the natural aquatic environments (Thelaus et al., 2008). The challenges lie in understanding interactions in complex and dynamic ecosystems, and further disentangling abiotic and biotic factors in large datasets.

Biotic interactions have been recognized as important for understanding the influence and functions of a community. Unfortunately, estimating such interactions in highly dimensional metabarcoding data is difficult, as the number of interactions grows exponentially with the number of taxa. Several efforts have been made to overcome this challenge, mostly based on defining the distance between assemblages, such as ordination or network methods (Faust and Raes, 2012; Weiss et al., 2016). However, these methods often include subjective choices and difficulties in interpreting the results. An appealing alternative is to *a priori* visualize a network for PRB based on direct acyclic graphs (DAG), where dependencies between taxa can be ordered in a topological sequence, which can be interpreted as taxa influencing other community members (e.g., A→B, taxa A leads to taxa B). In addition, recent advances within hierarchical modeling tools have paved the way toward using a joint model for abundance on taxa (underpinned by the generalized linear latent variable model generalized linear latent variable model (GLLVM); Warton et al., 2015; Niku et al., 2017; Ovaskainen et al., 2017). This multivariate tool can incorporate several aspects in a single analysis, such as assessing the impact of environmental factors on species abundance and finding a correlation between taxa. Still, a few studies have applied these tools for correlations in microbial ecology (e.g., Metcalf et al., 2016; Niku et al., 2017; Andersson et al., 2018).

To improve the understanding of the ecological niche and specific interactions between PRB and protozoa in aquatic systems, we performed a field study on the northern Baltic Sea coast. PRB were defined as bacteria that have been shown to resist amoeba degradation with varying ecological strategies (amoeba-associated and free-living). The target bacteria are based on the PRB identified by isolation from amoebas and bacteria shown to resist phagocytosis (Bertelli and Greub, 2012), listed in Table 1. We sampled four bays representing offshore water and river inflow during the productive season in the period from May to September. We subsequently combined the use of two statistical methods (DAG and GLLVM) in order to identify possible interactions between the phagotrophic protozoa and PRB. In addition, in order to identify the source for PRB, we used the source tracking method Random Forest (RF).

**TABLE 1:** Predation-resistant bacteria (PRB) identified by isolation from amoebas and bacteria shown to resist phagocytosis (Bertelli and Greub, 2012).

Phyla	Genus	PRB
*α proteobacteria*	*Afipia*	*Afipia felis, Afipia broomae*
*α proteobacteria*	*Bosea*	*Bosea spp.*
*α proteobacteria*	*Rickettsia*	*Rickettsia bellii*
*β proteobacteria*	*Burkholderia*	*Burkholderia cepacia, Burkholderia pseudomallei*
*γ proteobacteria*	*Coxiella*	*Coxiella burnetii*
*γ proteobacteria*	*Francisella*	*Francisella tularensis*
*γ proteobacteria*	*Pseudomonas*	*Pseudomonas aeruginosa*
*γ-proteobacteria*	*Legionella*	*Legionella drancourtii, Legionella longbeachae, Legionella pneumophila*
*Chlamydiae*	*Parachlamydia*	*Parachlamydia acanthamoebae, Protochlamydia amoebophila*
*Chlamydiae*	*Simkania*	*Simkania negevensis*
*Actinobacteria*	*Mycobacterium*	*Mycobacterium tuberculosis, Mycobacterium bovis, Mycobacterium avium, Mycobacterium smegmatis*
*Chlamydiae*	*Waddlia*	*Waddlia chondrophila*
*Bacteroidetes*	*Candidatus Amoebophilus*	*Amoebophilus asiaticus*

We hypothesized that in spring the freshwater inflow to the bays would seed and promote opportunistic EPRB, such as *Pseudomonas*. We expected IPRB, such as *Legionella*, to show strong links and dependency on certain protozoan hosts due to their intracellular lifestyle. These would increase in abundance later in the season, as protozoa often show maxima in summer.

## Results

### Environmental Conditions

The river inflow to the coastal area peaked in May due to the spring snowmelt in the catchment area (Supplementary Figure 1C). It was 35-fold higher in spring than in summer. This decreased the salinity in the bays, which had an influence on the bacterial composition in May (Supplementary Figure 1). The mean seawater temperature in the study bays [Valviken (VA), Kalvarskatan (KA), Stadsviken (ST), and Ängerån (AN)] and the offshore water (HÖRNE and ÖRE) was 10–12°C in spring, peaked in July (23–24°C), and decreased toward autumn (14°C) (Supplementary Figure 2). The salinity was lowest during the spring flood, ∼2.5 psu, and increased to 3.5–4 psu during the summer. The dissolved organic carbon (DOC) showed maximum values in spring and decreased as the season progressed (Supplementary Figure 2).

The chlorophyll a concentration and phytoplankton primary production peaked in spring and decreased in summer (Supplementary Figure 2). A similar trend was also observed for the protozoan biomass; however, occasional blooms of dinoflagellates were observed in summer (Supplementary Figure 3). The bacterial biomass was more stable over the study period (Supplementary Figure 4), showing slightly higher values in summer than in spring and autumn. In general, the bacterial production was higher at the innermost sampling site (position 1) than at the seaward sites in the bays (positions 2 and 3) (Supplementary Figure 2). The protozoan and bacterial biomass also tended to have higher values at the innermost position in the bays, decreasing in the seaward direction (Supplementary Figures 3, 4). Bacterial biomass showed lower values at the offshore sampling sites.

**FIGURE 1:**
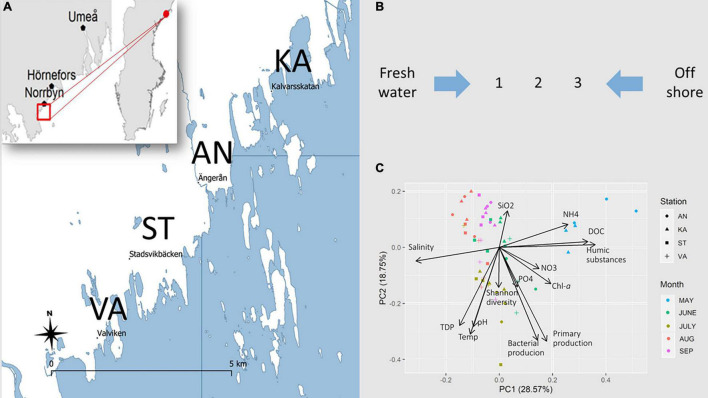
**(A)** Map of the studied coastal area in the northern Baltic Sea. Surface water samples were collected in four bays, Ängerån (AN), Kalvarskatan (KA), Stadsviken (ST), and Valviken (VA), throughout the productive season, May–September 2018. Additional samples were collected in freshwater inflows and offshore water. **(B)** In each bay, seawater was sampled at inner (1), middle (2), and outer (3) positions. **(C)** PCA of the spatiotemporal distribution of environmental variables in the study bays.

Principal component analysis (PCA) of environmental variables resulted in monthly clusters (Figure 1C), indicating that the temporal variation was larger than the spatial variation in the bays. The influence of DOC and humic substances were marked in May, while salinity influenced the distribution pattern in summer.

### Occurrence of Predation Resistant Bacteria (PRB)

**FIGURE 2:**
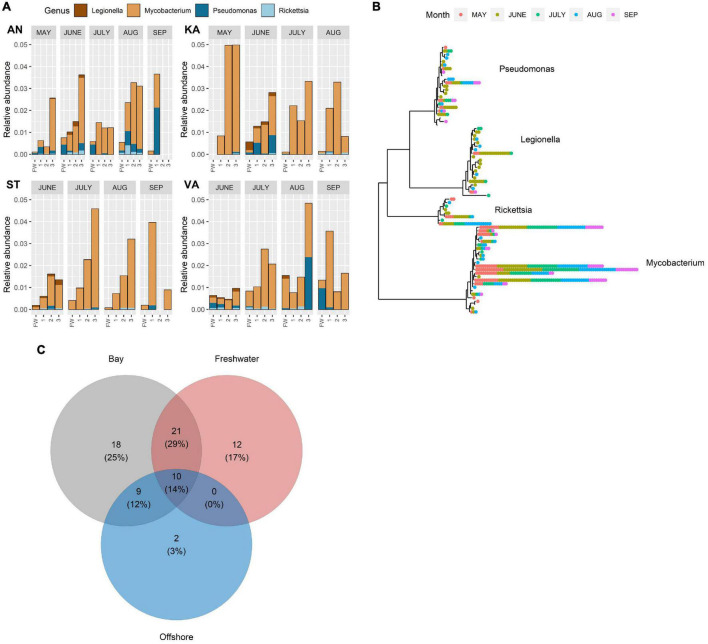
**(A)** Relative abundance of amplicon sequence variant reads (ASV) of different predation-resistant bacterial (PRB) genera in freshwater inflows and recipient bays (AN, KA, ST, and VA) during the study period (May–September). Sampling sites: FW (freshwater), 1 (inner bay), 2 (mid-bay), and 3 (outer bay). Note that samples were not collected in May in ST and VA or in KA in September. **(B)** Temporal variation of ASVs annotated to different PRB genera in the three water types. Each position in the radial tree represents an ASV. Colored dots denote different samples where the ASVs were detected. **(C)** Number of co-occurring and unique ASVs in freshwater, bays, and offshore water. Numbers within parentheses denote the ASV proportion of the entire PRB community.

**FIGURE 3:**
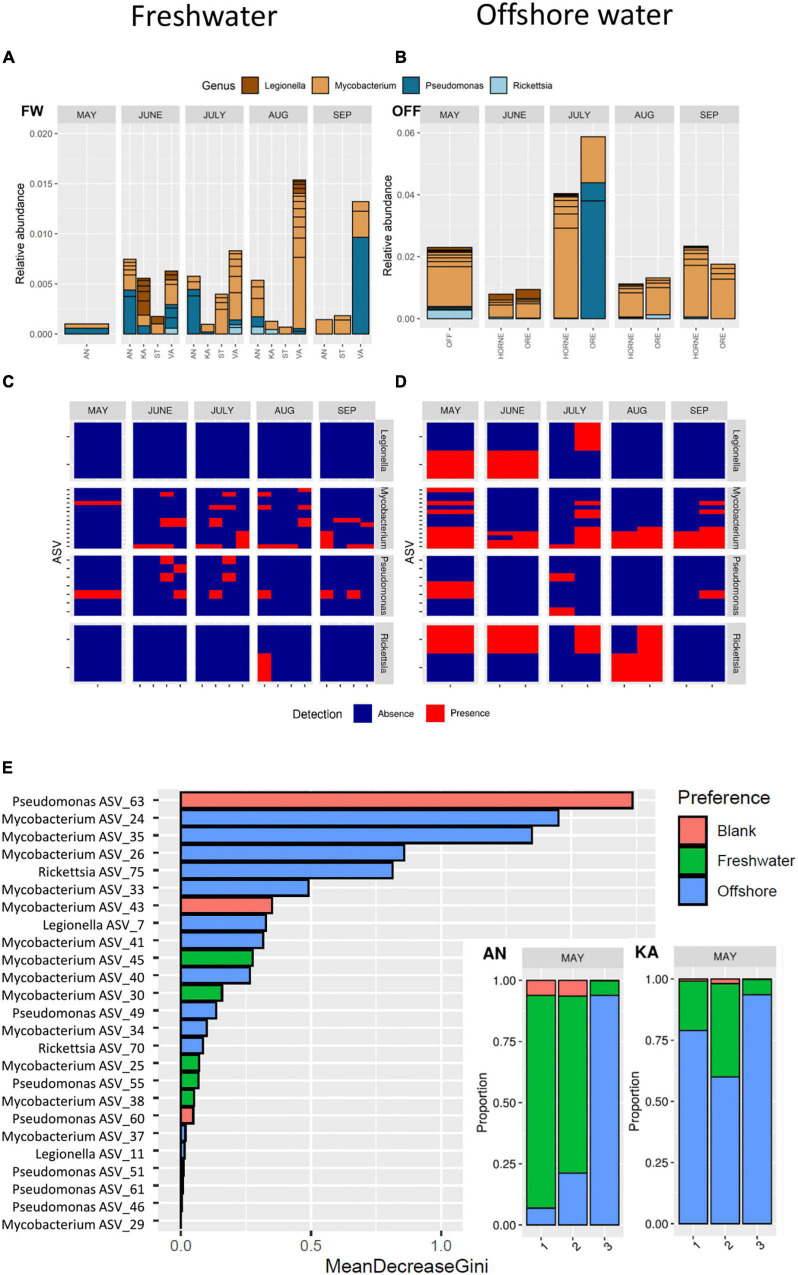
Top: Predation-resistant bacteria in freshwater **(A)** and offshore water **(B)** assigned at the genus level from May to September. Middle: Presence and absence of each amplicon sequence variant (ASVs detected in ≥4 samples) from May to September for freshwater **(C)** and offshore water **(D)**. Bottom: Random Forest analysis of the source predicted for each PRB ASV, sorted by variable importance **(E)**. The variable importance (mean decrease gini) for each ASV is highlighted by the source (Red: Blank, Green: Freshwater, or Blue: Offshore). For the bays AN and KA in May, the proportion of each source is presented.

Predation-resistant bacteria were detected in all the collected samples, that is, in freshwater, bays, and offshore water throughout the study period (Figures 2A, 3). A total of 76 amplicon sequence variants (ASVs) were identified, which were distributed to four PRB genera. Several families that contain PRB were also detected, however, without further taxonomic information (Supplementary Figure 5). The majority of the PRB genera corresponded to *Mycobacterium* (25 ASVs), followed by *Pseudomonas* (23 ASVs), *Legionella* (20 ASVs), and *Rickettsia* (8 ASVs) (Figure 2B). The relative abundance of the different genera approximately followed the PRB ASV distribution, where *Mycobacterium* was the most abundant PRB and *Rickettsia* was the least abundant (Figures 2A, 3A).

Some seasonal trends of the PRB abundances were observed. *Legionella* was detected mainly in June, when it showed widespread occurrence in freshwater, the bays, and offshore water (Figures 2, 3). *Rickettsia* occurred scarcely and seem close to the detection limit in September (Figures 2, 3). Within a month, the PRB composition showed a somewhat similar distribution pattern from the inner to the outer parts of the bays, even though variations were also found (Figure 2A). Seasonal trends of different ASVs within the same genus were also found (Figure 2B). For example, some ASVs of *Pseudomonas*, *Mycobacteria*, and *Rickettsia* occurred only in May (Figure 2B).

The number of ASVs, that is, the sequence diversity, was about twice as high in the bays and freshwater than observed in the offshore water (Figure 2C). Approximately, 30% of the ASVs were shared among freshwater and bays, while only 10% were shared among all the three habitats. A total of 25, 17, and 3% of the ASVs were unique for the bays, freshwater, and offshore water, respectively. The number of *Legionella* ASVs was higher in freshwater and in the bays compared to offshore water (Supplementary Table 1). For the other PRB genera, no difference in the number of ASVs could be detected between the habitats.

Analysis of the absence/presence of widespread ASVs detected in ≥4 samples showed that *Mycobacterium* and *Pseudomonas* occurred in both freshwater and offshore water all through the study period (Figures 3C,D). In contrast, ASVs of *Legionella* and *Rickettsia* mainly occurred in offshore water during the spring–summer period and not in autumn (September) (Figures 3C,D).

Random Forest analysis indicated that specific ASVs belonging to *Mycobacterium*, *Rickettsia*, *Legionella*, and *Pseudomonas* originate from offshore water, while specific ASVs belonging to *Mycobacterium*, *Rickettsia*, and *Pseudomonas* originate from freshwater (Figure 3E and Supplementary Figure 8). In spring, as much as 90% of the PRB ASVs at the innermost station of AN were predicted to originate from freshwater, while the freshwater influence decreased markedly in a seaward direction. In the bay receiving less river inflow, the analysis indicated that ASVs at all sampling sites in KA to a higher degree originated from the offshore water. Thus, the potential freshwater origin of many PRB was notable, such as for certain ASVs of *Mycobacterium* and *Pseudomonas* (Figure 3E). Thus, the spring river inflow was identified as significant source habitat for many of the PRB ASVs.

### Phagotrophic Protozoa Resolved at the Species Level

We designed a novel set of primers specific for 18S rRNA amplicon sequencing, based on the V6F and V8R regions. Based on our *in silico* primer predictions, this design has the benefit of both increasing the taxonomic resolution and covering much of the diversity. The benefits of this region are shown in detail by Latz et al. (2022). Unlike the 18S rRNA V9 region, this longer 18S rRNA V6-V8 region was able to assign the majority of the taxa to species level (>50%). For the relative abundance of protozoans (Supplementary Figure 7, 30 most abundant taxa), Ciliophora (ciliates) was most abundant over the season. The relative abundance for dinoflagellates was the highest in May in all bays. Telonemia mostly occurred in August and September, while Ochrophyta was detected all through the season. Similar to PRB, we found a taxonomic difference over the season, as well as between the bays, for example, Apicomplexa were only detected in Valviken. However, the variability in composition was great between the bays and freshwater in most cases, with species of the family Strobilidiiae only present in freshwater. A highly similar composition of protozoa was observed for all sampling positions (1, 2, and 3) in each bay, indicating little within-bay variation of protozoa and good reproducibility.

### DAG Analysis Identifies PRB That Are Dependent on Protozoans

**FIGURE 4:**
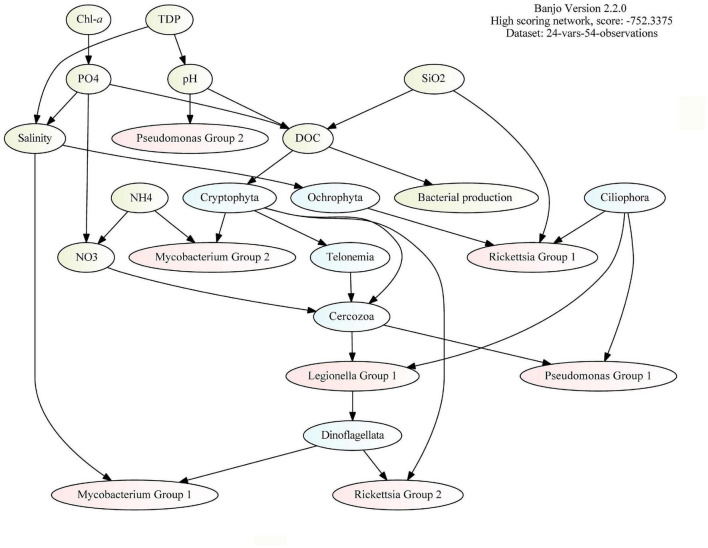
Direct acyclic graph (DAG) analysis (by BANJO) of environmental data (green), 16S rRNA ASVs (red), and 18S rRNA ASVs (blue). The bacterial ASVs (red) are grouped by clustering (by k-means clustering based on pairwise Pearson correlations between ASV abundance within the genus) and the protozoan ASVs (blue) are grouped by division.

To infer interactions between bacteria and protozoa in this coastal area, we merged three datasets (based on environmental data found in Figure 1C and relative abundance data found in Figure 2A and Supplementary Figure 7) to construct a DAG network. Ecotypes within genus were identified by clustering the ASVs into groups (Group 1 and Group 2), based on the relative abundance in the samples. According to the model (Figure 4), the most important connection in the PRB network (according to influence score statistics) is the dependency of bacterial production on dissolved organic carbon (Supplementary Table 2). Overall, in the inferred network, the PRB are directly dependent on the protozoans. PRB ASVs assigned to *Rickettsia*, *Legionella*, and *Pseudomonas* are dependent on the relative abundance of Ciliophora ASVs, which suggests that these organisms interact with each other.

**FIGURE 5:**
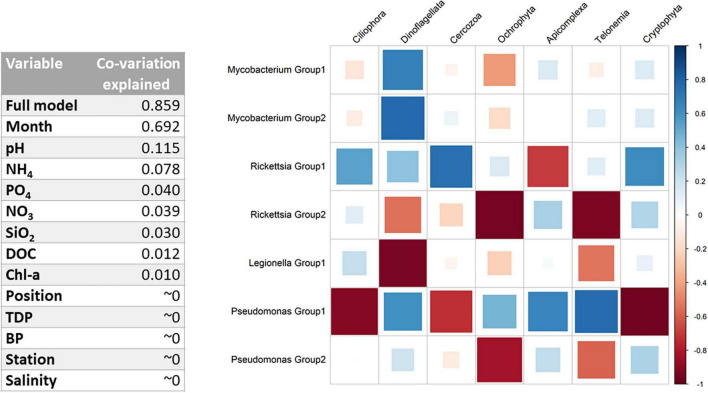
**(Left)** Co-variation explained in the amplicon sequence variant (ASV) communities for each environmental variable (according to the ratio of traces). The full model corresponds to the following variables: Position*Month + Station*Month + Position*Station + pH + NH_4_ + PO_4_ + DOC + Salinity. **(Right)** Full-model GLLVMs estimated taxa correlations after accounting for environmental or spatiotemporal effects.

### GLLVMs Analysis Identifies Interactions Between PRB and Protozoa

To better understand co-occurrence patterns, we need to disentangle the environmental factors and interactions identified by DAG, while correcting for spatiotemporal effects (Figures 1C, 2A). The independent contribution of each independent variable in the model is found in Figure 5, Left, as much as 69.2% and the full model explains 85.9% of the total co-variation in the PRB and protozoan groups. For the full model, combination effects of the month, position (1, 2, and 3), and bay (i.e., station) were included to account for specific conditions. Other environmental variables used in the model were pH, NH_4_, PO_4_, DOC, bacterial production, and salinity. After the inclusion of the predictors in the full model, the seasonal variations were captured, allowing predictions of biotic interactions (Supplementary Figure 10B). Significant parameters for each PRB group are found in the coefficient plot in Supplementary Figure 5 and Supplementary Table 5. For both *Pseudomonas* and *Rickettsia*, strong differences between the within-genus PRB groups were inferred, with opposite effects of most predictors (in 22 and 33 out of total 41 cases, respectively), suggesting niche differentiation within these genera. This effect was not as pronounced for *Mycobacterium*, suggesting that this group is more homogeneous. Furthermore, many combinations of predictor months, bay, and position were significantly pointing toward strong local adaptation, at least for *Pseudomonas* and *Rickettsia*. *Legionella* was significantly overrepresented in June and in bay VA.

The remaining 14.1% of the co-variation in the model not explained after the inclusion of predictors was likely to be explained by biological interactions. After accounting for environmental and spatiotemporal effects, the GLLVMs identify interactions between PRB and protozoa, such as between *Legionella* and Ciliophora, and between *Rickettsia* and Ciliophora (Figure 5, Right). *Mycobacterium* and Dinoflagellata show a strong positive correlation, while *Legionella* and Dinoflagellata show a strong negative correlation. Members of *Pseudomonas* Group 1 show a strong correlation with all phagotrophic protozoa, either positively or negatively. PRB–protozoa interactions are therefore an important source of variation.

## Discussion

In this study, ecological niches and specific interactions between PRB and protozoa were identified in a coastal area in the northern Baltic Sea. Four public health-relevant PRB genera, *Mycobacterium, Legionella, Pseudomonas*, and *Rickettsia*, were found to occur throughout the productive season in the study area. Information about protozoan diversity was obtained by designing and applying a new set of V6–V8 18S rRNA-specific primers for amplicon sequencing. Specific ASVs of the PRB co-occurred with certain phagotrophic protozoa in the bays, freshwater discharge, and offshore water. The PRB occurrence and PRB–protozoa associations depended strongly on the time and place of sampling. By applying GLLVMs and direct acyclic graphs (DAGs), we were able to examine biological relationships beyond temporal and local effects. One important result from the analysis was the identification of a link between *Legionella* and ciliates (Ciliophora) in June, possibly due to an endosymbiotic relationship as *Legionella* has an intracellular lifestyle (Gomez-Valero and Buchrieser, 2019).

### PRB Occurrence Is Influenced by Strong Spatiotemporal Effects

In general, a strong spatiotemporal variation of PRB abundance was inferred by our model, where the month of sampling and interacting variables between month, station, and position was most important in explaining the PRB occurrence (Figure 5 left and Supplementary Figure 5). Interestingly, the location was of importance even though the geographic scale was relatively small (∼8.5 km between bays KA and VA), indicating local adaptions and habitat fragmentation. Further, we found unique ASVs at different sampling sites (Figure 2B), which also support PRB local adaptation.

Amplicon sequence variants specific to *Legionella*, in particular, displayed a strong seasonality as they occurred more or less exclusively during June in all bays. Previous studies have suggested that temperatures above 20°C promote the relative abundance of *Legionellales* in freshwater (Graells et al., 2018). Herein, we observed a peak of *Legionella* in June when the water temperature was around 12°C, which did not differ significantly from May (Supplementary Figure 2). Generally, for the Baltic Sea, the south–north differences in temperature and the salinity gradient make the northern parts more similar to freshwater. During an inventory of bacterial 16S rRNA amplicon sequences along the salinity gradient of the Baltic Sea, Herlemann et al. (2011). identified marked differences in the bacterial community compositions of brackish water compared to freshwater and marine counterparts (Herlemann et al., 2011). Taken together, the northern parts hold an easier habitat to exploit by species adapted to freshwater, such as *Legionella*. The low salinity in the study area may explain why *Vibrio* was not detected in this study (Baker-Austin et al., 2012), even though this genus has been reported in the Baltic Proper (Gyraite et al., 2019). Considering climate change, and the predicted effect on both temperature and salinity, the Baltic Sea may face different challenges in the future (Andersson et al., 2015).

Freshwater is considered the major reservoir for the pathogenic *Legionella pneumophila* (Boamah et al., 2017). However, *Legionella* is often studied in man-made water systems, such as cooling towers (Brigmon et al., 2020), due to the thermophily of the pathogen *L. pneumophila* (Lesnik et al., 2015). Even though it is known that *Legionella* exist in freshwater and have cold-adapted variants (Lesnik et al., 2015), few studies have reported their occurrence in natural aquatic environments, such as in lakes, rivers, and estuaries (Carvalho et al., 2007; Parthuisot et al., 2010; Shimada et al., 2020). However, a large meta-analysis of the obligate intracellular family Legionellales (also including *Rickettsia*) shows that these bacteria exist in half of the freshwater and marine water samples globally (Graells et al., 2018). Importantly, that study found that both cold and marine waters contain many uncultured variants, as confirmed by our study. To summarize, we could not identify temperature as a driver for the occurrence of *Legionella* in this area. Rather, the occurrence was inferred by biotic interactions.

### DAG and GLLVM Identify PRB Preferences to Protozoa

In order to infer biotic interactions based on environmental parameters and sequence data, it is necessary to disentangle factors causing spatial and temporal variations. By using two complementary inference methods, interactions were predicted between specific PRB and protozoa, such as between *Mycobacterium* and Dinoflagellata, between *Legionella* and Ciliophora, and between *Rickettsia* and both Ciliophora and Cercozoa (Figures 4, 5). Since the statistical models are a simplification of a complex dynamic ecosystem, it is worth noting that missing significant predictors can have an effect on the results. Therefore, it is possible that not all correlations represent true biotic interactions. This study focused on bacteria that have previously shown resistance to degradation by a protozoan, which does not guarantee resistance to other protozoans. The mentioned interactions are, however, supported by previous works, which have reported endosymbiosis of *L. pneumophila* in the ciliate *Paramecium caudatum* (Watanabe and Watarai, 2017; Nishida et al., 2018; Watanabe et al., 2018) and association of a *Rickettsia*-like bacterium with another common ciliate (Vannini et al., 2005; Ferrantini et al., 2009). In addition, Szokoli et al. (2016), reported the association of two Rickettsiales symbionts with the ciliate protist *Paramecium biaurelia* via the cytoplasm. Rickettsiales members have also been associated with Cercozoa species, where Hess et al. (2016) discovered endosymbionts in an ameboflagellate. Also, several species of *Mycobacterium* have previously been shown to replicate inside the dinoflagellate *Dictyostelium discoideum* (Solomon et al., 2003; Steinert and Heuner, 2005).

The mentioned studies isolated the bacteria and protozoa to identify their interactions. This requires that the organisms can be cultivated, which is often a major challenge considering intracellular bacteria (Singh et al., 2013). As an alternative approach, Lamrabet et al. (2012) used genomic information to search for protein homologs within *Mycobacterium* and three protozoans (*D. discoideum* included), which support the exchange of genetic material. For inferring novel interactions, we show that our approach identified potentially similar interactions at the community level in the natural environments of both PRBs and protozoa. Thus, our approach could be a viable option to detect novel interacting microbes in their natural habitats using culture-independent methods. Isolation and single-cell genomics could then be used to further investigate potential interactions (Boscaro et al., 2022).

### Managing Rare Sequence Variants and Avoiding Bias

The PRB identified occurred at relatively low abundances in the sequence data, constituting a maximum of 3–5% of the entire bacterial community. A common pattern for *Legionella, Mycobacteria*, and *Rickettsia* ASVs was the detection of rare sequence variants that only occurred on a few occasions or few sampling sites (≤4 samples). In line with previous studies (Niku et al., 2017; Roguet et al., 2018), the rare sequence variants were not included in the models (DAG, GLLVM, and Random Forest), since they increase the noise and risk for false positives. Thus, 54 rare sequence variants were excluded (out of 76 in total), of which many occurred in freshwater (Supplementary Table 4). The resulting model that includes the widespread ASVs shows a generally increased detection frequency of these ASVs in offshore waters (Figures 3A–D). This was supported in the Random Forest analysis that predicted the source for each ASV (Figure 3E), where offshore water was the dominating habitat, except during the spring flood in May for AN that was receiving the largest amount of river inflow (Figure 3E). To summarize, even after the exclusion of rare sequence variants, Random Forest was able to identify a gradient without the knowledge of the study design.

### PRB Diverse in Freshwater – Abundant in Offshore Water

In general, the sequential diversity of PRB was higher in freshwater than in the marine system, where each ASV was detected in fewer freshwater samples compared to the ASVs found in the offshore samples. However, the relative abundance of the widespread ASVs was higher in the offshore water. *Pseudomonas* and *Mycobacterium* were abundant during the spring river flush in the AN bay which has the highest freshwater inflow. The number of *Pseudomonas* ASVs detected in freshwater was larger when compared to offshore water, but the difference was not statistically significant (n = 9 for freshwater and n = 4 for offshore). The GLLVM analysis indicated that *Pseudomonas* and *Mycobacterium* were adapted to several local environments (Supplementary Figure 9 and Supplementary Table 5), and based on the source tracking analysis, different ASVs seemed to originate from different sources (Figure 3E). The model predicted that certain *Pseudomonas* and *Mycobacterium* ASVs originated from freshwater, especially in AN bay during spring (Figure 3E). Importantly, it seems possible for *Pseudomonas* to originate from the spring flood and survive during the season, as could be the case for one of the ASVs seen in Figure 3C.

*Pseudomonas* is a ubiquitous genus occurring worldwide in many environments, for example, in soils, freshwater, estuaries, and marine systems (Hutalle-Schmelzer and Grossart, 2009; Lindh et al., 2015; Pent et al., 2017; Qiao et al., 2018). *Pseudomonas* is opportunistic and able to benefit from disturbed or polluted conditions (Andersson et al., 2018; Rizzo et al., 2019). These bacteria have previously been described in the Baltic Sea, and some can cause infections in fish and other animals (Lönnstrom et al., 1994; Wiklund, 2016; Sonne et al., 2020). Alarming detection of *Pseudomonas* has been previously described after disturbing events, such as flooding (Shankar et al., 2021), and in wastewater effluents (Luczkiewicz et al., 2015). Our results suggest that the spring flood was a disturbance for the bacterial community (Supplementary Figure 1) and indicate that *Pseudomonas* profited by quickly exploiting resources during the spring colonization of the bays.

### PRB: A Diverse Group With Varying Co-occurrences

The model clustering of the ASVs indicated that the bacteria constituted different PRB ecotypes. We identified different protozoan host preferences within the PRB genus, wherein two particular ASVs assigned to *Rickettsia* demonstrated varying co-occurrences (Figure 5, Right). These conflicting patterns apply to both the protozoan interactions and association with location and environmental variables, indicating different niche preferences. In addition, the two groups of *Pseudomonas* showed many strong negative correlations with protozoans (Figure 5, Right). These findings are consistent with previous studies where highly resolved sequence clusters showed opposite environmental preferences across a gradient of disturbance (Newton and McLellan, 2015) and over a gradient of salinity (Herlemann et al., 2011). Both *Pseudomonas* and *Rickettsia* are heterogeneous groups of bacteria including not only human, animal, and plant intracellular pathogens, but also non-pathogenic free-living relatives. In addition, variation in traits, such as biofilm formation, is expected in these genera. Several strains of *Pseudomonas* are known to produce toxins (Matz et al., 2004), some of which can kill protozoans, such as amoebas (Matz et al., 2008). Therefore, the many strong negative correlations between *Pseudomonas* Group 1 and, for example, Ciliophora and Cercozoa could be interpreted as excretion of toxins harmful to potential grazers. Thus, when studying interactions between organisms in the genus containing potential human pathogens, it is important to account for genetic variability within the genus and host preference.

## Conclusion

We conclude that PRB constitute a group of highly diverse bacteria with many unique sequence variants that are hard to predict. Our data suggest that freshwater, coastal water, and offshore water pose a risk of harboring potentially pathogenic bacteria. We observe strong spatial and temporal variation in the occurrence of PRB. Amplicon sequence variants of *Pseudomonas*, *Rickettsia*, *Legionella*, and *Mycobacterium* were more diverse in freshwater, while more abundant in seawater, and occurred at specific periods throughout the productive season. These dependencies prevent investigators from inferring specific interactions that depend on biotic interactions, such as with protozoans. By applying two statistical models, we were able to disentangle interactions in a complex and dynamic ecosystem. We showed that the PRB have varying co-occurrences and host preferences. For example, our model suggests that the maximum population of *Legionella* recorded in June was dependent on the occurrence of Ciliophora. Thus, this work provides a basis for identifying sources and drivers for potential human pathogens that survive in the environment.

## Materials and Methods

### Study Sites

From May to September 2018, monthly water samples were collected at 0.5 m depth at three sampling points in four bays along the northern Baltic coast: Ängerån (AN), Kalvarsskatan (KA), Stadsviken (ST), and Valviken (VA) (Figure 1A). Additionally, samples were taken from the freshwater inlets (FW) and offshore waters: Örefjärden (ORE), Hörnefors (HORNE), and Degerfjärden (OFF). The first sampling coincided with the spring snowmelt, and the bays were thus exposed to relatively high freshwater inflow in May than during later samplings. Due to the unstable ice situation, we were not able to sample Stadsviken and Valviken bays in May. Ängerån (AN) received the largest river discharge among the sites and was the brownest in water color (Figure 1C), while Kalvarsskatan (KA) had no river inflow and was relatively a clear water bay. Stadsviken (ST) and Valviken (VA) received intermediate freshwater inputs compared to AN and KA. In total, 80 water samples were collected. Therefore, our sites covered a gradient in freshwater supply and associated inputs of terrestrial dissolved organic matter and nutrients. The magnitude of this gradient varies temporally. Procedures for measurement of physicochemical variables, primary production, bacterial production, and microscopic analysis of phytoplankton are presented in Supplementary Materials and Methods.

### Bacterial DNA Sampling and Extraction

At each sampling occasion, 200-500 ml of water was gently filtered (≤20 kPa) onto 0.2-μm filters (Pall Coporation sterilized filters, Supor^®^ 0.2 μm, 47 mm, S-pack white gridded). For freshwater samples, <500 ml was filtered due to limitations associated with the filtering of turbid water. A total of 80 water samples were collected. The filters were then folded using cleaned tweezers and placed in 2-ml Eppendorf tubes. The samples were stored at –80°C until the DNA extraction. Filters were thawed and placed into PowerWater bead beating tubes. The DNA was extracted using a DNeasy^®^ PowerWater kit^®^ (Qiagen, Hilden, Germany) according to a modified DNeasy PowerWater protocol. The samples were treated with an additional heating step (horizontal water bath for 30 min, 65°C) and with a bead beating step in each direction of 20 Hz for 3 × 3 min with a Tissuelyser II (Qiagen, Hilden, Germany). Two filter blanks were used as a negative control during DNA extraction. When the DNA extracts were obtained from the DNeasy^®^ PowerWater^®^ kit, the samples were prepared for PCR analysis by Illustra columns (MicroSpin S-200 HR, GE Healthcare). The final DNA was frozen before subjecting to PCR analysis. Sample preparation, thermal cycling, and PCR product preparation were performed in separate rooms.

### 18S rRNA Primer Design

In order to design a suitable candidate PCR primer pair to target conserved regions in the 18S rRNA (Hugerth et al., 2014), which covers a large taxonomic coverage, we used the program Primer Prospector (Walters et al., 2011). The primer pairs V6F: 5′- AATTYGAHTCAACRCGGG-3′ (Hadziavdic et al., 2014) and V8R: 5′-GACRGGCGGTGTGNACAA-3′ (Edgcomb et al., 2011) were evaluated, using higher degeneracies than earlier publications. To predict the taxonomic coverage, PR2 version 4.12.0 (Guillou et al., 2012) and SILVA (Pruesse et al., 2007) databases were used. All major taxonomic divisions were covered (Supplementary Figure 11). The forward and reverse primers were modified to incorporate a 12-bp Golay error-correcting barcode that enables sample multiplexing (Caporaso et al., 2012). The primers were optimized and tested for secondary structures (Gibbs free energy: mean, –11 kcal/mol).

### Amplicon Preparation and Sequencing

For the 16S rRNA amplicon preparation, DNA was amplified and sequenced as previously described (Hägglund et al., 2018), apart from using the PCR purification kit. In short, the DNA was amplified using the No. 5 Hot Mastermix 2.5x kit (5 PRIME, Qiagen, Hilden, Germany) with bacteria/archaeal primers 515F/806R specific for the hypervariable V4 region of the 16S rRNA gene. The forward and reverse primers were modified to incorporate a 12-bp Golay error-correcting barcode that enables sample multiplexing (Caporaso et al., 2012). All samples were amplified in triplets and pooled after PCR amplification (94°C for 3 min, followed by 35 cycles at 94°C for 45 s, 50°C for 1 min, 72°C for 1.5 min, and finally 10 min rest to finish).

For the 18S rRNA amplicon preparation, DNA was amplified using the No. 5 Hot Mastermix 2.5x kit (5 PRIME, Qiagen, Hilden, Germany) with eukaryotic primers V6F/V8R (V6F: 5′-AATTYGAHTCAACRCGGG-3′ and V8R: 5′-GACRGGCGGTGTGNACAA-3′) specific for the hypervariable V6–V8 region of the 18S rRNA gene. All samples were amplified in triplets and pooled after PCR amplification (94°C for 3 min, followed by 35 cycles at 94°C for 45 s, 57°C for 1 min, 72°C for 1.5 min, and finally, 10 min rest to finish).

For PCR, three template blank reactions were used by using water instead of template. The PCR product was run on a 1% agarose gel, and the DNA concentration was estimated with a Qubit fluorometer (Invitrogen, Carlsbad, CA, United States). The amplicons were pooled at equimolar concentrations and purified with the QIAquick PCR purification kit (Qiagen, Hilden, Germany) following the supplier’s instructions. The DNA concentration of the pooled amplicon product was measured with a Qubit fluorometer and adjusted to 2 nM. The library was denatured and diluted as described by Illumina (MiSeq System User Guide, Part # 15027617 Rev. C), before it was loaded onto a MiSeq cartridge (Illumina, San Diego, CA, United States) and sequenced using a 2 × 300 bp paired-end sequencing protocol (Hägglund et al., 2018).

### Quality Control and Raw Data Processing

Demultiplexing was done using deML (Renaud et al., 2015). The Quantitative Insights Into Microbial Ecology (QIIME2) pipeline, version 2019.1, was used for processing raw sequence data (Bolyen et al., 2019). Greengenes version 13.8 (McDonald et al., 2012) and PR2 version 4.12.0 (Guillou et al., 2012) databases were used for bacteria and eukaryote taxonomic assignment. Quality filtering was done using dada2 default parameter values. For the 16S rRNA sequence raw data processing, the reads were trimmed to 220 bp for the forward read and to 65 bp for the reverse read. For the 18S rRNA sequence raw data processing, the reads were trimmed to 280 bp for the forward read and to 170 bp for the reverse read, allowing a maximum of 5 and 8 expected errors per read, respectively.

### Target Organisms

Since a diverse range of bacteria has been shown to use various strategies for predation resistance, the dataset was first analyzed toward a set of known PRBs (Bertelli and Greub, 2012; Table 1). Phylogenies were estimated based on FastTree 2 (Price et al., 2010). To test whether a relationship existed between sampling environment (i.e., bays, offshore, and freshwater) and PRB detection frequency, we made use of Fisher’s exact test (Supplementary Table 1) implemented in the R statistical software.

In order to include protozoa in the analysis based on knowledge of feeding style, including heterotrophs and mixotrophs, other phylogenetic groups were excluded from the eukaryotic dataset (Olenina et al., 2006). The supergroup Archaeplastida was excluded. For the supergroup Opisthokonta, Choanoflagellates were retained as they are filter feeders (Richter and Nitsche, 2017), while the Divison of Metazoa and Fungi were excluded. For the supergroup Stramenopiles, heterotrophic and mixotrophic Chrysophyceae were included, since they have phagotrophic feeding styles. From the Division Ochrophyta, classes Chrysophyceae, Dictyochophyceae, and NA were retained, while non-relevant classes were excluded (Bolidophyceae, Bacillariophyta, MOCH-2, Phaeothamniophyceae, MOCH-5 Raphidophyceae, Synurophyceae, Eustigmatophyceae, Phaeophyceae, and Xanthophyceae). The sequence data were visualized using the R package phyloseq (McMurdie and Holmes, 2013) and ggplot2 (Wickham, 2016).

### Microbial Source Tracking of PRB

The dataset with PRB bacterial taxa and phagotrophic protists was adjusted to the requirements of the analysis. To find the source of specific PRBs, we created a subset by filtering out taxa that did not meet the criteria of detection in a minimum of four samples. To assign PRB ASVs to their source environments, the Random Forest (RF) classification method implemented in the RandomForest R package was used (Liaw and Wiener, 2002). The source environments (i.e., training data) were defined as offshore (n = 9 samples), freshwater of river inflow (n = 17), and template-free PCR water (n = 7), with presence/absence data of PRB ASVs in all samples included. RF models were tuned using 10-fold cross-validation with a grid search of parameters to optimize the predictive performance: ntree 500, 1,000, 2,000, 5,000 (number of trees in the forest), mtry 1–10 (number of candidates drawn to feed the algorithm), and maxnodes 5–15 (set the maximum amount of terminal nodes in the forest). The tuning procedure was performed with R packages caret (Kuhn, 2008) and e1071 (Meyer et al., 2020).

### Direct Acyclic Graphs—BANJO

Dynamic Bayesian Network (DBN) was used to model interactions between PRB bacterial taxa and phagotrophic protists. The dataset with PRB bacterial taxa and phagotrophic protists with detection in a minimum of four samples was adjusted to the requirements of the analysis. First, the data were normalized such that the count of each sample was equal to the data (to compensate for the difference in the total number of reads between the samples). To account for within-group variation (such as niche differences), a subset of PRB and phagotrophic protozoa were gathered, respectively (Supplementary Table 3, 4). The DBN was created by collapsing the sequence datasets to the highest taxonomic resolution possible, 16S rRNA ASV table to genus, and 18S rRNA ASV data to species. The data were also collapsed by filtering out taxa that did not meet the criteria of detection in a minimum of four samples. Selected environmental variables were also included to infer dependencies (edges) between bacteria, protists, and environmental factors (nodes), and thus, potential drivers of community structure. Then, all features (i.e., bacteria and protists groups, and environmental variables) were normalized such that the mean value was centered at zero and the variation was transformed to one to be able to compare across datasets. For phagotrophic protists, the top 30 most abundant ASVs were then grouped to division. PRB ASVs were clustered using k-means clustering in R based on pairwise Pearson correlations between ASV abundance within the genus. For each genus, two partitions were chosen with the exception of the genus *Legionella*, which did not show any conflicting ASV abundance pattern.

The DBN was inferred (Smith et al., 2002) using BANJO version 2.2.0^1^ (for an application of the use of BANJO in the community, see Metcalf et al., 2016) using the following analysis conditions: discretization = 2 intervals, max parents = 3, min lag = 0, max lag = 0, and Simulated Annealing search algorithm. These parameter choices were based on preliminary runs minimizing the negative log-likelihood score (i.e., resulted in best goodness-of-fit to the data). To aid the best fit and a logic network, environmental nodes were only permitted as parent nodes. Also, no edges between the bacteria were permitted. For other settings, default parameters were used.

### Generalized Linear Latent Variable Models (GLLVMs)

A generalized linear latent variable model was used to model interactions between PRB bacterial taxa and phagotrophic protists. The dataset was adjusted to the requirements of the analysis in the same way as for the DBN analysis with both 16S rRNA ASV clusters within genus and 18S rRNA ASVs clustered at the species levels comprising the outcome data. The GLLVM was constructed using the R package gllvm (Niku et al., 2019). Gaussian distribution function (i.e., identity link) for the responses was selected. The following environmental variables were included in the model as continuous predictors: pH, DOC, phosphate (PO4), salinity, chlorophyll a concentration (Chl-a), and ammonium (NH4). To avoid including highly correlated variables as predictors in the model, a pre-selection step was performed based on the PCA score plot of the environmental variables (i.e., only one of a group of tightly clustered variables was selected). The selected variables were normalized so that the mean value and standard deviation were set to zero and one, respectively, for each included predictor. Design variables included were as follows: station (i = 1,…,4), month of sampling (j = 5,…,9), and the position of the sampling point in each bay (k = 1,2,3). Second-order interactions between all design variables were included, as these interactions greatly improved the goodness-of-fit of the model to the data. To improve convergence, jitter variance for starting values of latent variables was set to 0.5, and the number of initial runs was set to five. Three latent variables were selected to capture the residual variation following recommendations suggested in a previous study (Warton et al., 2015). Otherwise, default values of the GLLVMs function were used.

## Data Availability Statement

The datasets presented in this study can be found in online repositories. The names of the repository/repositories and accession number(s) can be found in the article/Supplementary Material. Supplementary data related to this article can be accessed at the Short Read Archive (https://www.ncbi.nlm.nih.gov/sra), project accession: PRJNA796828, ID: 796828.

## Author Contributions

KE processed the samples for sequencing. KE and JA processed the data after sequencing and conducted data analyses. KE wrote the manuscript together with all co-authors. AA, JT, and JA organized the data collection and supervised the project. AA supervised the data collection. All authors contributed to the article and approved the submitted version.

## Conflict of Interest

The authors declare that the research was conducted in the absence of any commercial or financial relationships that could be construed as a potential conflict of interest.

## Publisher’s Note

All claims expressed in this article are solely those of the authors and do not necessarily represent those of their affiliated organizations, or those of the publisher, the editors and the reviewers. Any product that may be evaluated in this article, or claim that may be made by its manufacturer, is not guaranteed or endorsed by the publisher.

## Footnotes

1
http://www.cs.duke.edu/~amink/software/banjo/
