# Supplementary material for: Microbial Interactions — Underexplored Links Between Public Health Relevant Bacteria and Protozoa in Coastal Environments
Source: Front Microbiol. 2022 Jun 13;13:877483. doi: 10.3389/fmicb.2022.877483 (PMC9235517; doi:10.3389/fmicb.2022.877483)
Supplement: Supplementary file 1 [file Data_Sheet_1.docx]

**Supplementary Material 1**

**Supplementary material and methods**

**Experimental procedure - Physicochemical variables**

Salinity and water temperature were measured at 0.5 m below surface with a WTW ProfiLine Cond 3110 conductivity-meter. For chemical measurements, water samples were collected at 0.5 m depth and filtered through Supor membrane syringe filters (0.2 µm pore size, non-pyrogenic, Acrodisc®, Pall). Concentrations of dissolved inorganic phosphorus (DIP, i.e. phosphate PO_4_), dissolved inorganic nitrogen (DIN, i.e. nitrate, nitrite and ammonium) and silicate (SiO_2_) of the samples were analysed using the Seal QuAAtro39 autoanalyser (Grasshoff et al. 2007). Samples for total dissolved nitrogen (TDN) and Total Dissolved Phosphorus (TDP) were analysed in the same way as the inorganic nutrients. Water samples for DOC concentration measurements were also filtered, the filtrates were then acidified (18 mM HCl, final concentration) and analysed using a Shimadzu TOC-5000.

The concentration of humic substances (HS) was measured from unfiltered water samples using a Perkin Elmer LS 30 fluorometer at 350/450 nm excitation/emission wavelengths, calibrated with a serial dilution of quinine sulphate solution (Hoge et al. 1993).

Water bearing data [m³/s] available from the public database of SMHI, station Torrböle 2, stream Öreälven, (63.7017°, 19.6037°) where used for information about the spring flood.

**Experimental procedure - Chlorophyll a and primary production**

Samples for Chl *a* (100 ml) were filtrated onto 25 mm GF/F filters under low pressure and stored at -80°C until analysis. Chl *a* were extracted in 95% ethanol in the dark overnight at 4ºC. Samples were centrifuged for 10 minutes to separate ethanol containing chlorophyll *a* from solid material. The concentration of chlorophyll *a* was measured with a Perkin Elmer LS 30 fluorometer (433 nm excitation and 674 nm emission wavelength).

*In situ* photosynthetic rates of phytoplankton were measured using the ^14^C incorporation method. 5 ml of seawater were placed in four 20 ml bottles (three light and one dark) and incubated *in situ* with 7.2 µl ^14^C (^14^C Centralen Denmark, activity 100 μCi ml^-1^) for a minimum of 3 hours. Post incubation, 100 µl of 5 M hydrochloric acid were added to each tube and samples were ventilated for 12 hours. 15 ml of scintillation cocktail were added to each sample and samples were measured on a Beckman 6500 scintillation counter. Dissolved inorganic carbon was calculated based on temperature, pH and salinity according to Gargas (1976). Daily net primary production (PP) was calculated using the “light factor method” as described in (Gargas et al. 1976, Andersson et al. 1996).

**Experimental procedure - Bacterial production**

The ^3^H-thymidine incorporation method was used to measure bacterial production (BP) (Fuhrman and Azam, 1982). Triplicate 1 ml seawater samples (one control and two samples) were incubated with 2 μl of ^3^H-thymidine (84 Ci mmol^-1^; PerkinElmer, Massachusetts, USA) (final concentration 24 nM) for 1 hour at *in situ* temperature. This thymidine addition corresponded to the saturation level. The control sample was pre-killed by adding 100 μl of ice-cold 50 % TCA and incubation at -20°C for 5 minutes. Cell production was calculated using a conversion factor of 1.4×10^18^ cells mol^−1^ of incorporated thymidine (Wikner and Hagström 1999). Daily net production rates were calculated assuming stable uptake rates over the day and a bacterial carbon content of 20 fgC cell^-1^ (Andersson et al. 2018).

**Experimental procedure - Microscopy of protozoa**

Protist samples were collected from 0.5 m depth. Samples were fixed with Lugols solution and 10 ml were settled overnight in a sedimentation chamber. Protozoa were then analysed in an inverted microscope using the Utermöhl method and phase contrast (Utermöhl 1958). Taxa were identified and enumerated. The size of the cells was measured and their biovolume calculated (Olenina et al. 2006). Carbon biomass was calculated using biovolume to carbon biomass conversion factors (Menden-Deuer and Lessard 2000). This microscopic method is constrained to nano- (2-20 µm) and micro scale (20-200 µm), and thus picoeukaryotes (<2 µm) are not included in the analyses. The estimated biomass of phagotrophic protozoa are presented in *Supplementary Figure S2*.

**Experimental procedure - Microbial source tracking (MST) of whole bacterial community**

To investigate the influence of the freshwater inflow and the (brackish) offshore water on the bay assemblage compositions, a MST analysis was performed using the software SourceTracker, version 1.0.0. To reduce the required computational time, all assemblages were rarefied to a total sequencing depth of 10,000. Default parameters for controlling A) the Markov chains (i.e. convergence) and B) for prior specification of ASVs abundance in the different environments.

The MST library was defined using three environments: (i) no water communities represented (n = 7 representing communities); (ii) freshwater communities selected by freshwater stations (n = 17); and (iii) offshore water communities selected by offshore water stations (n = 9). The freshwater and offshore environments contained communities sampled across the study period at all available sites to account for seasonal and spatial variation. PCR samples with no template was included to track possible contamination from the laboratory (Pullerits et al. 2020).

The no template PCR samples (i), the freshwater samples (ii) and the offshore stations (iii) were in this way each denoted as different “sources” or environments. As “sinks”, samples collected at the sampling points one, two and three, respectively, at each bay was used (*Figure 1B*).

**Freshwater-offshore influence on salinity in the bays**

The freshwater influence on salinity in the bays was calculated according to:

(Fw-B)/ (Fw-O) *(Figure S1B)*

Fw= freshwater value, B= bay value, O= Offshore value

**Supplementary results**

**Microbial Source Tracking (MST) show freshwater influence on bacterial community in May**

To understand the influence of freshwater and offshore water on the bacterial communities in the bays, a source tracking analysis was performed. The freshwater samples, the offshore stations and the no template PCR samples (i.e. representatives of possible contamination from the laboratory) were each denoted as different “sources”.

The MST show that most samples indicate an ASV profile more similar to the offshore profile compared to the freshwater profile (*Figure S1A*). However, some samples indicated a microbial signature some similar to the source of freshwater, these samples are primarily from May from station AN and KA. The station AN receives the largest amount of freshwater inflow, which influences the bacterial community especially in May. The same trend can be observed for the freshwater influence (Supplementary *Figure S1B*), calculated from salinity measurements. These findings are consistent with the spring flood reported in May (Supplementary *Figure S1C*).

**Bacterial families that include PRB**

Even if the predation resistance is not always certain at a family basis, analysis on this taxonomic level can still be relevant for the understanding of the natural presence, sources and drivers for the occurrence of PRB in the coastal area. Spatial and seasonal trends for the occurrence of these families were found, e.g. for the bays KA and VA, *Coxiellaceae* was detected mainly in the freshwater samples (Supplementary, *Figure S5*). Regarding the diversity of these families, multiple ASVs were found for *Coxiellaceae, Legionellaceae, Mycobacteriaceae, Parachlamydiaceae, Rickettsiaceae and Pseudomonadaceae.* Even though the ASVs where were detected throughout the season, a few ASVs dominate for each family and could be detected in multiple samples (Supplementary, *Figure S6*).

**Challenges when studying low abundant bacteria**

When studying low abundant taxa it is important to remember that lack of detection does not necessarily mean that the bacteria is absent. For example, the genus level of *Rickettsia* seem to be on the edge of detection, especially in September (*Figure 2A*). For the freshwater samples, that had increased amounts of DOC and humic substances, less water was possible to filter for DNA sample preparation. Therefore, it is possible that there is a bias in the lower relative abundance of PRBs found in these samples. Bacteria close to the edge of detection (such as *Rickettsia*) is more prone to be effected by such a bias. However, the GLLVMs coefficients plot does indicate a positive correlation for *Rickettsia* group 2 in September (*Supplementary Figure S9, Table S5*), demonstrating the power with this tool also for low abundant taxa.

**Supplementary figures**


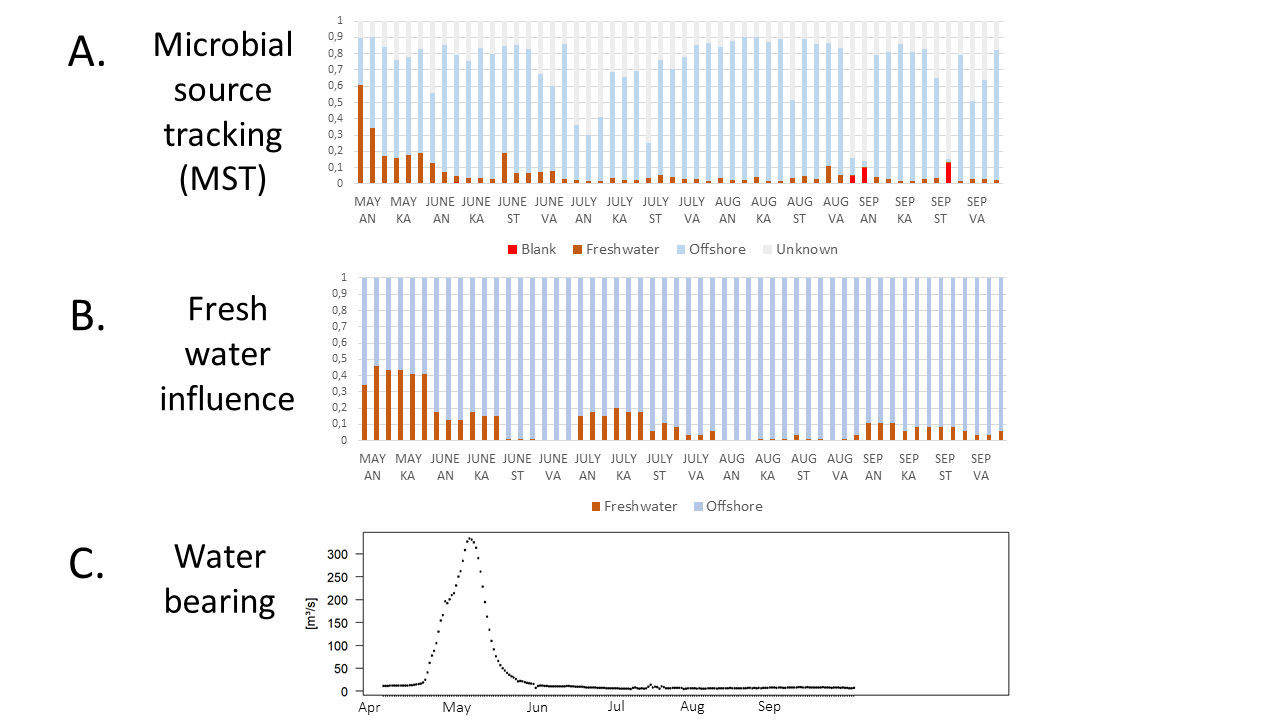


Figure S1: A: Microbial source tracking (MST) of all bacterial ASVs: Freshwater samples, the offshore stations and the PCR blanks were each denoted as different “sources”. The unknown profile is corresponding to the ASVs that were unable to be assigned to any of the sources. B: Fresh water influence, calculated from salinity measurements. C: Water bearing data [m³/s] available from the public database of SMHI, station Torrböle 2, stream Öreälven, (63.7017°, 19.6037°), providing information regarding the spring flood. The spring flood started around the 19th of April and ended in the end of May 2018.


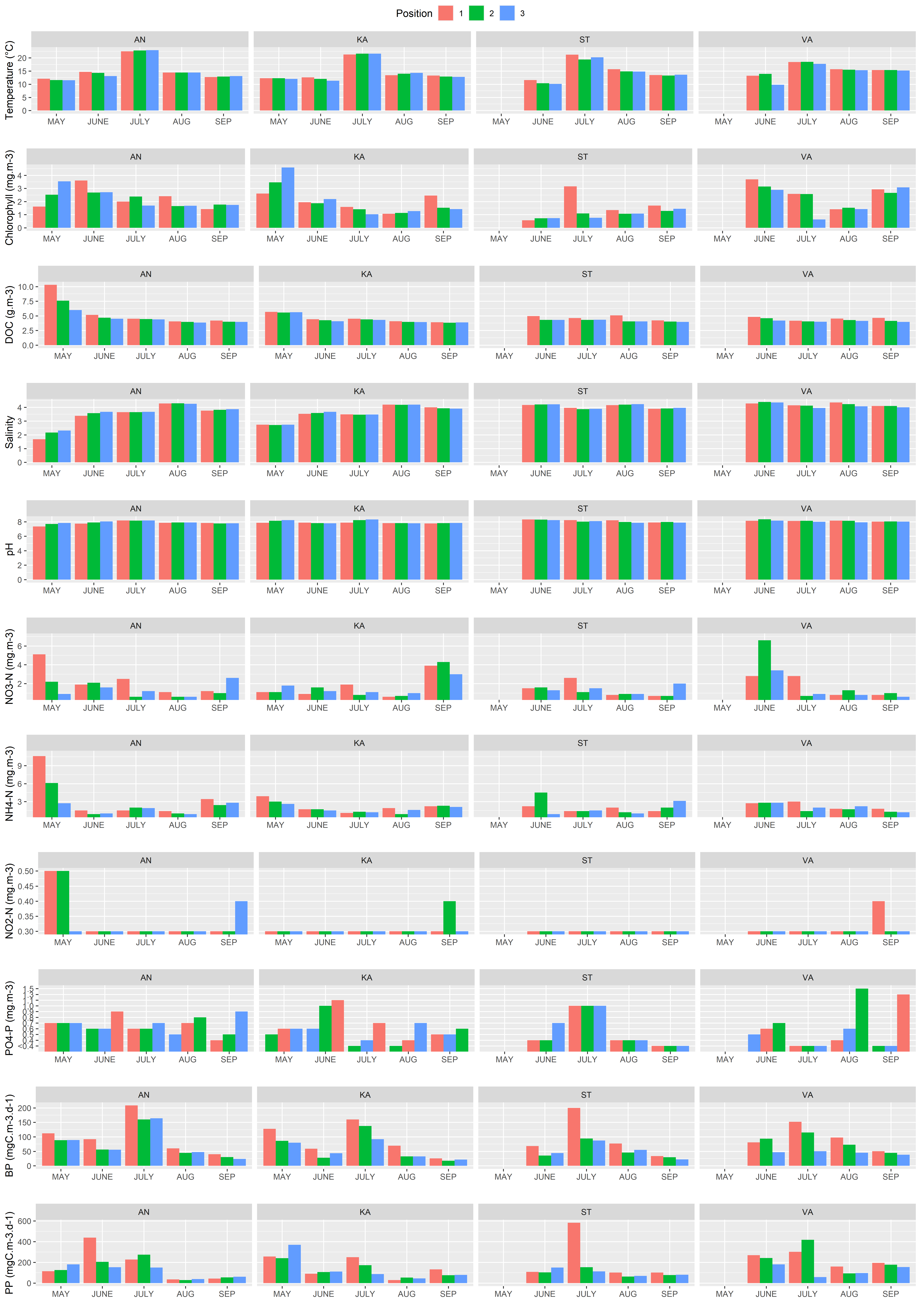


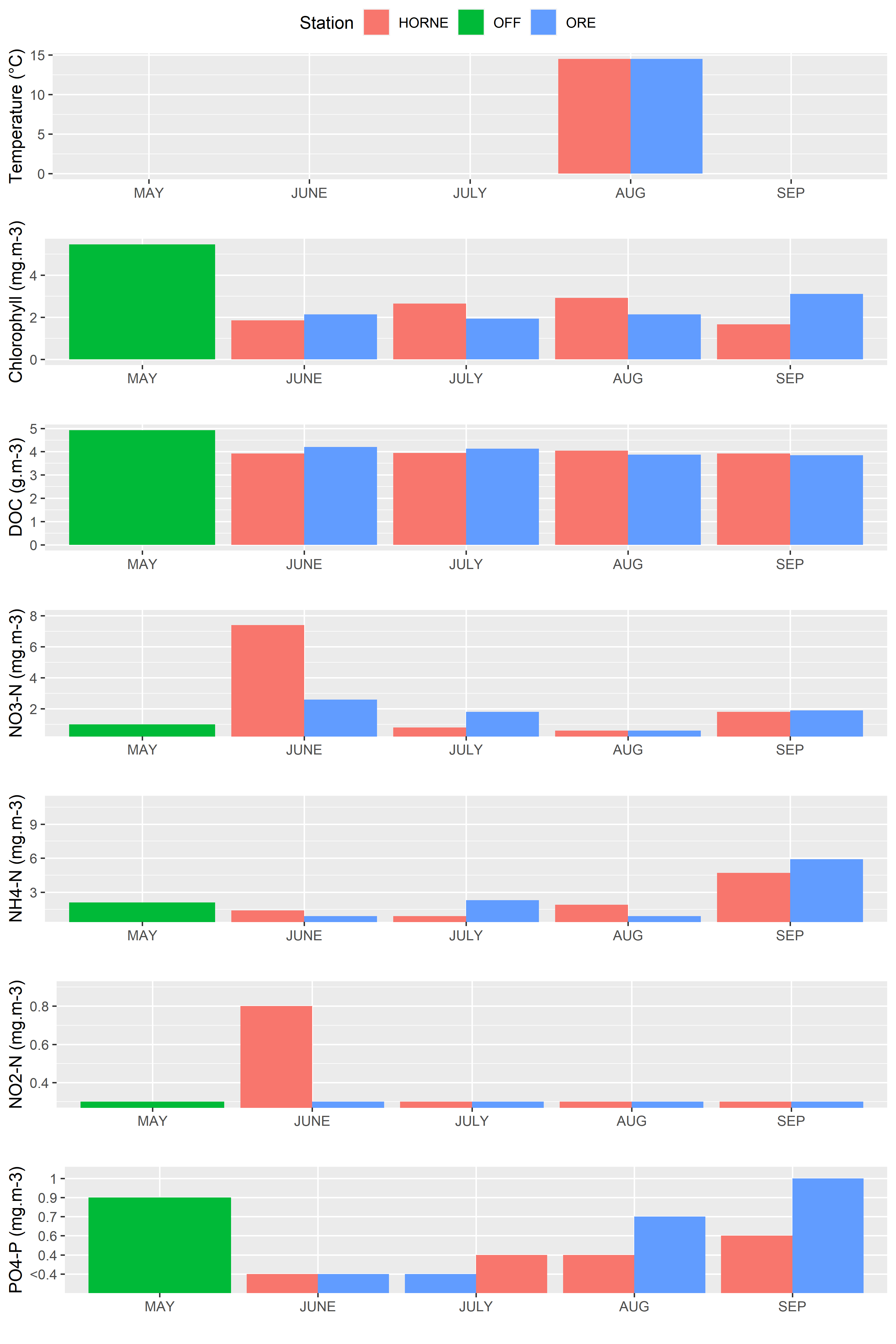


Figure S2: Environmental data sorted by month for the 4 bays (top) and for the three offshore stations (bottom).


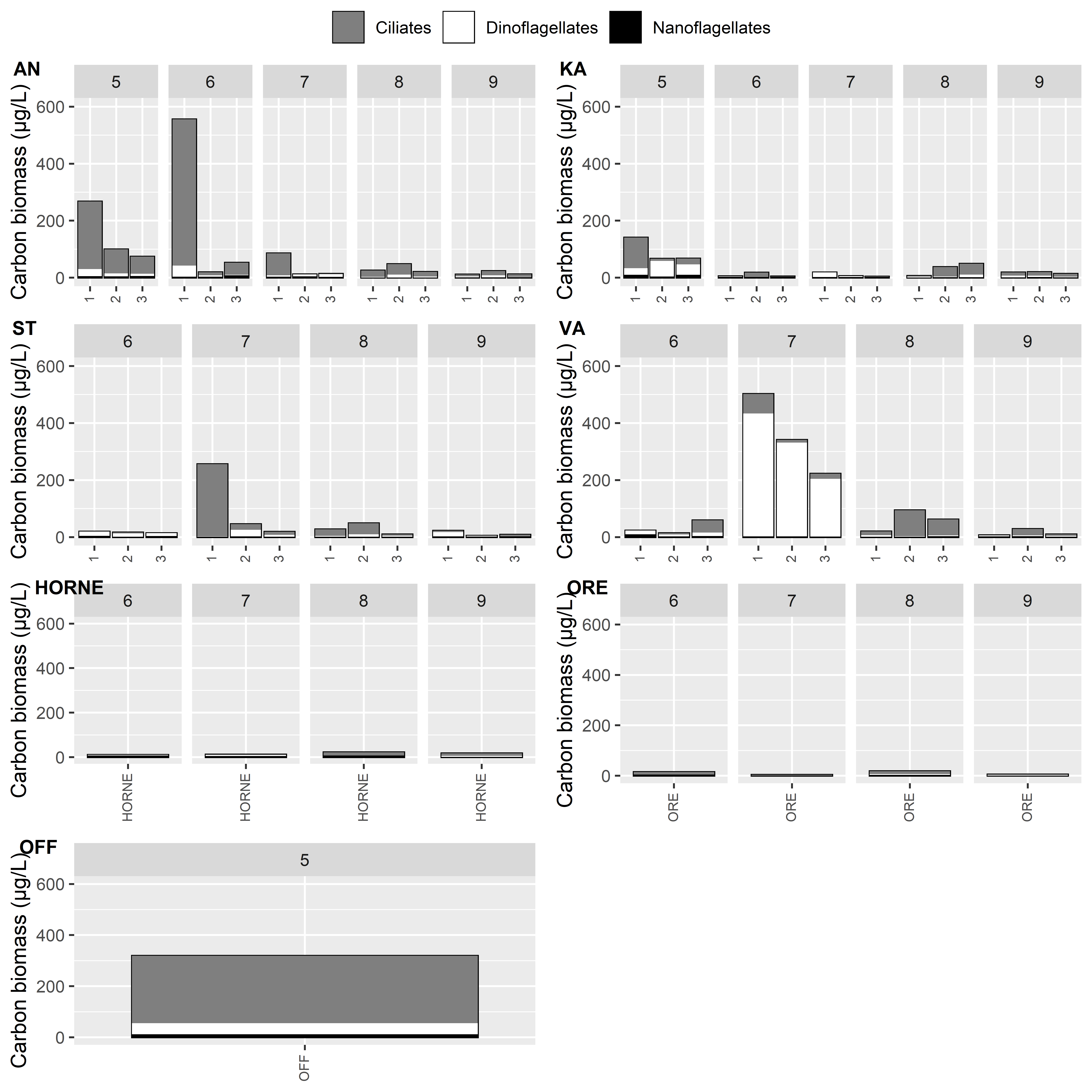


Figure S3: Carbon biomass concentrations of phagotrophic eukaryotes in the study bays (KA, ST, AN, VA) and offshore stations (HORNE, ORE, OFF) , analyzed by microscopy. Ciliate, dinoflagellate and nanoflagellate data are presented at position 1, 2 and 3 in the four bays during different months of the year (5-9). Organisms in group Ciliates include Ciliates sp., Favella sp., Leegardiella ovalis, Strombidium acutum, Strombidium vestitium, Tinntinopsis sp. and Vorticella spp. Organisms in group Nanoflagellates include Flagellates sp, Telonema subtile, Katablepharis sp. and Paraphysomonas spp. Organisms in group Dinoflagellates include Amphidinium sp., Dinophyceae spp., Ebria tripartita, Gymnodiniales and Protoperidinium bipes.


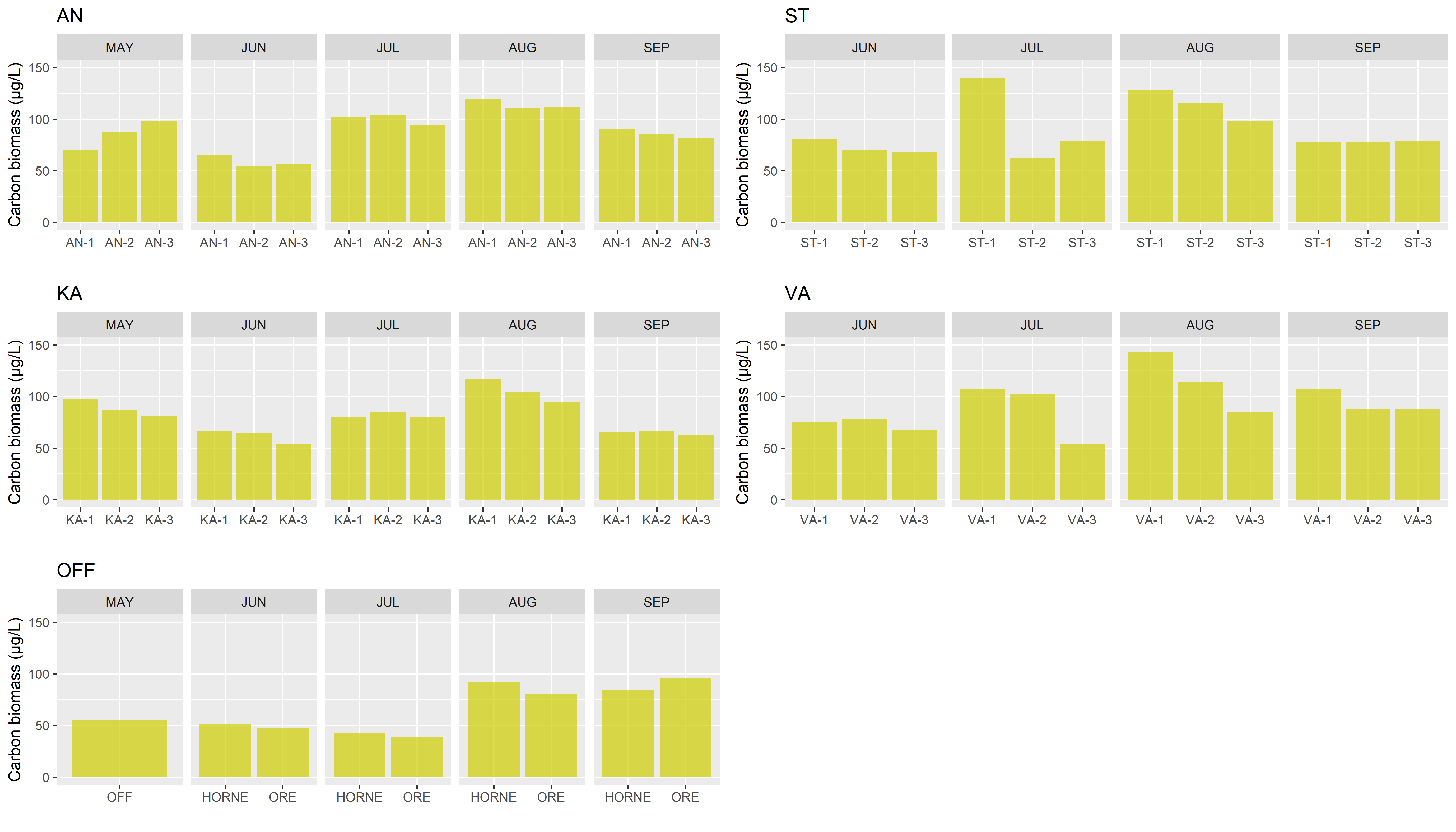


Figure S4: Bacterial carbon biomass concentrations at position 1(inner), 2 (mid) and 3 (outer) in the bays and at the offshore sampling sites during different months.


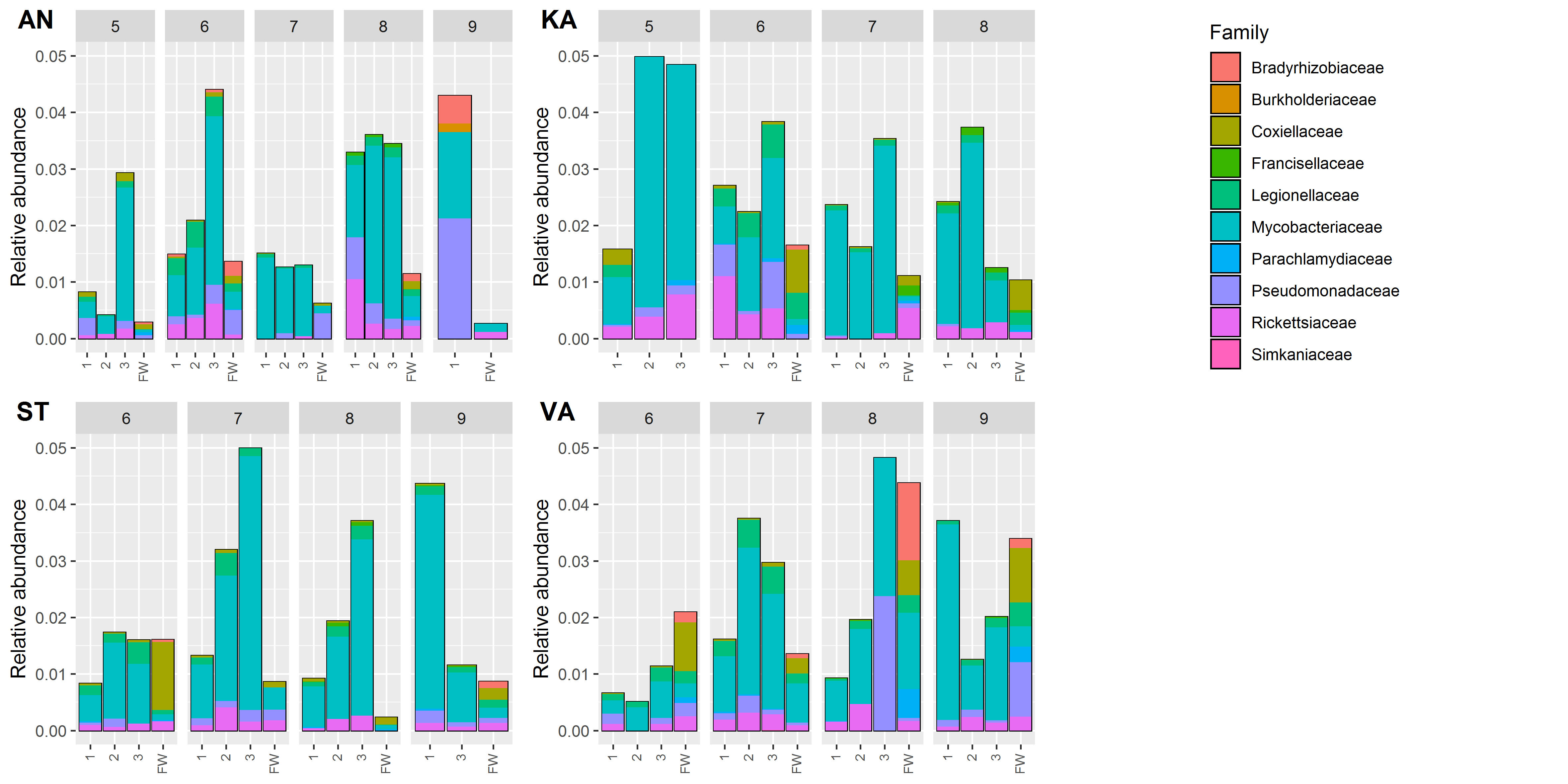

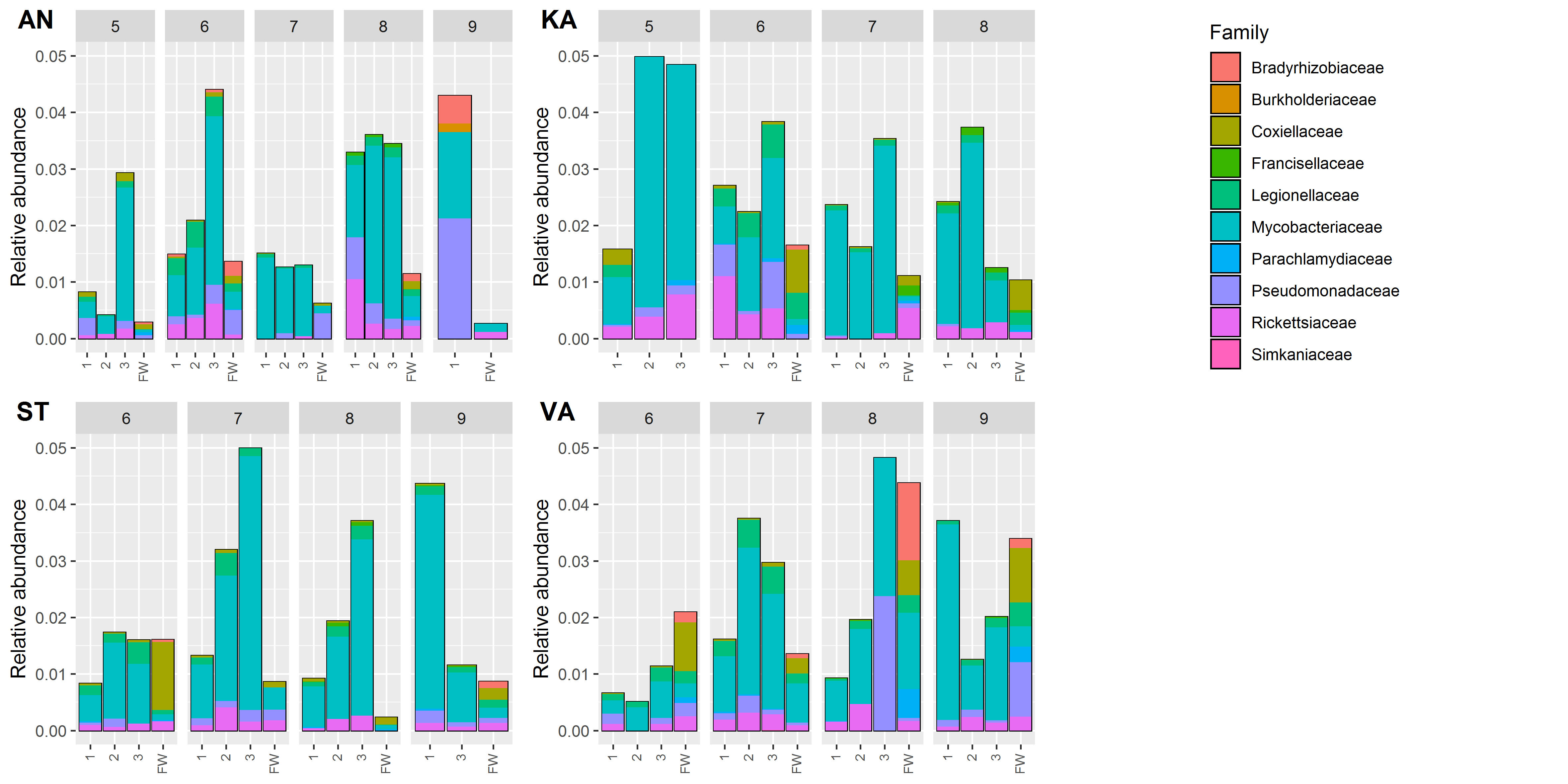


Figure S5: Predation resistant bacteria (PRB) assigned at the family level in the four bays (AN, KA, ST and VA) during different months of the year (5-9). X-axis denote sampling position 1 (inner), 2 (mid) and 3 (outer) in the bays, and freshwater sampling.

**
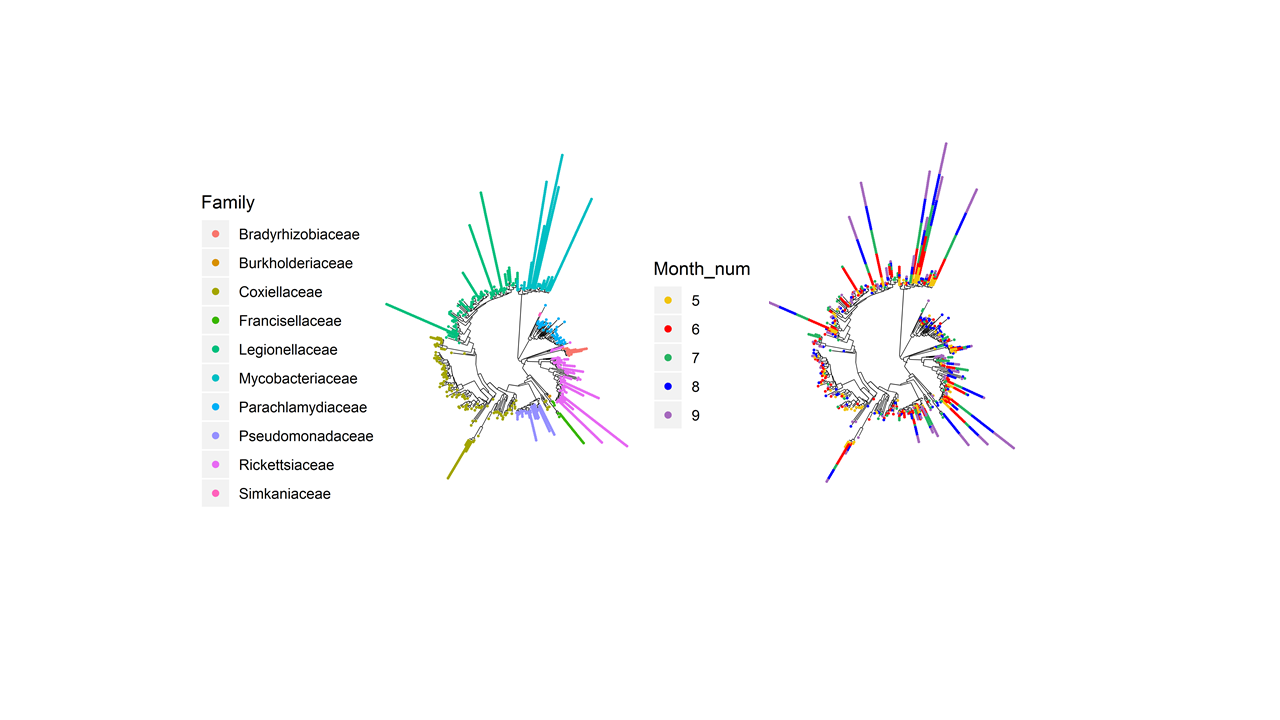
**

Figure S6: Occurrence of Predation resistant bacteria (PRB) taxonomically assigned at family level. Radial trees of ASVs colored by family (left) and by temporal detection (right).


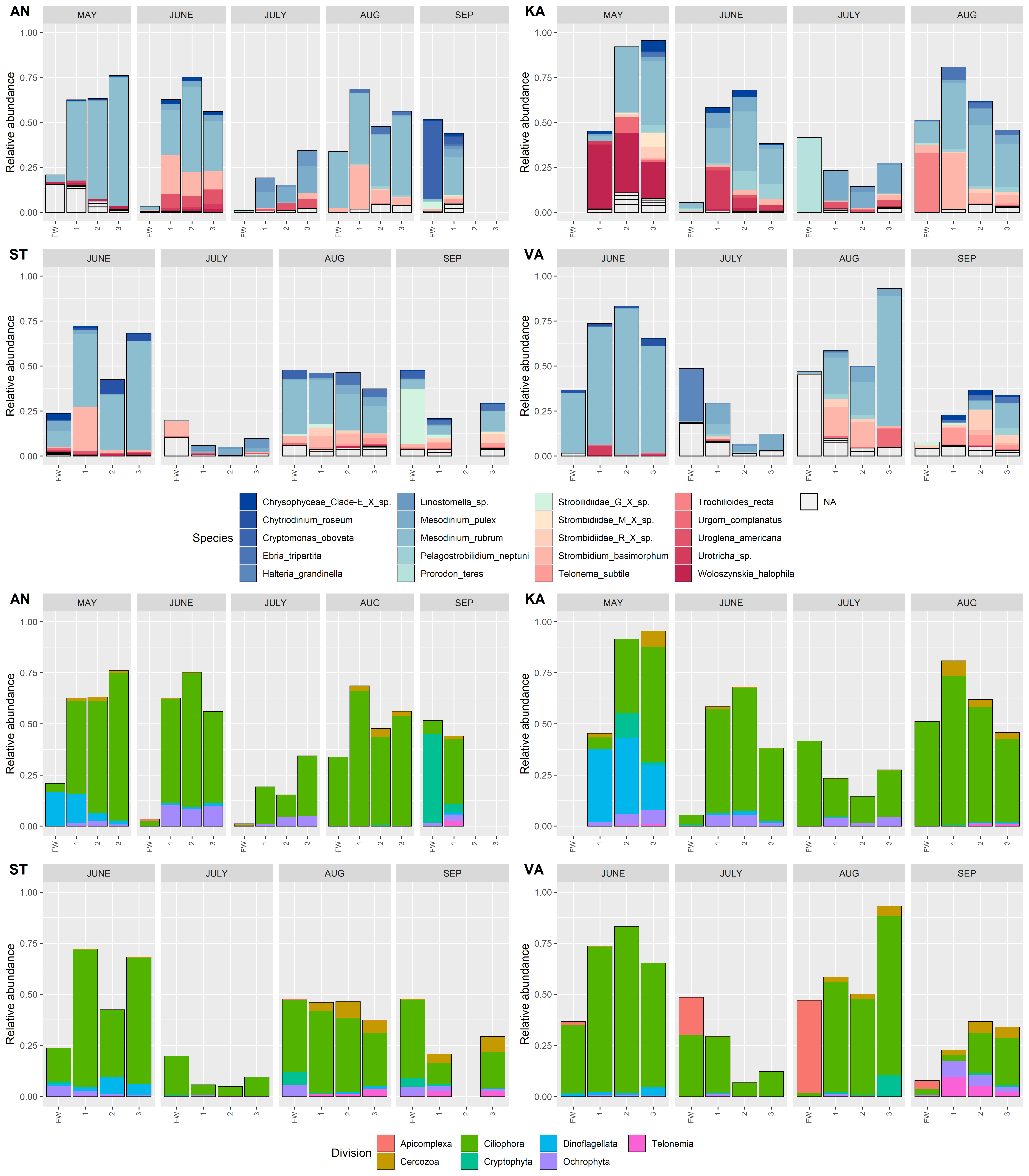


Figure S7: Relative abundance of phagotrophic protozoa in the four bays (KA, ST, AN, VA), analyzed by 18 S sequencing. The top 30 most abundant taxa are presented. Top two rows: Relative abundance of different species. Bottom two rows: Relative abundance of different Divisions.


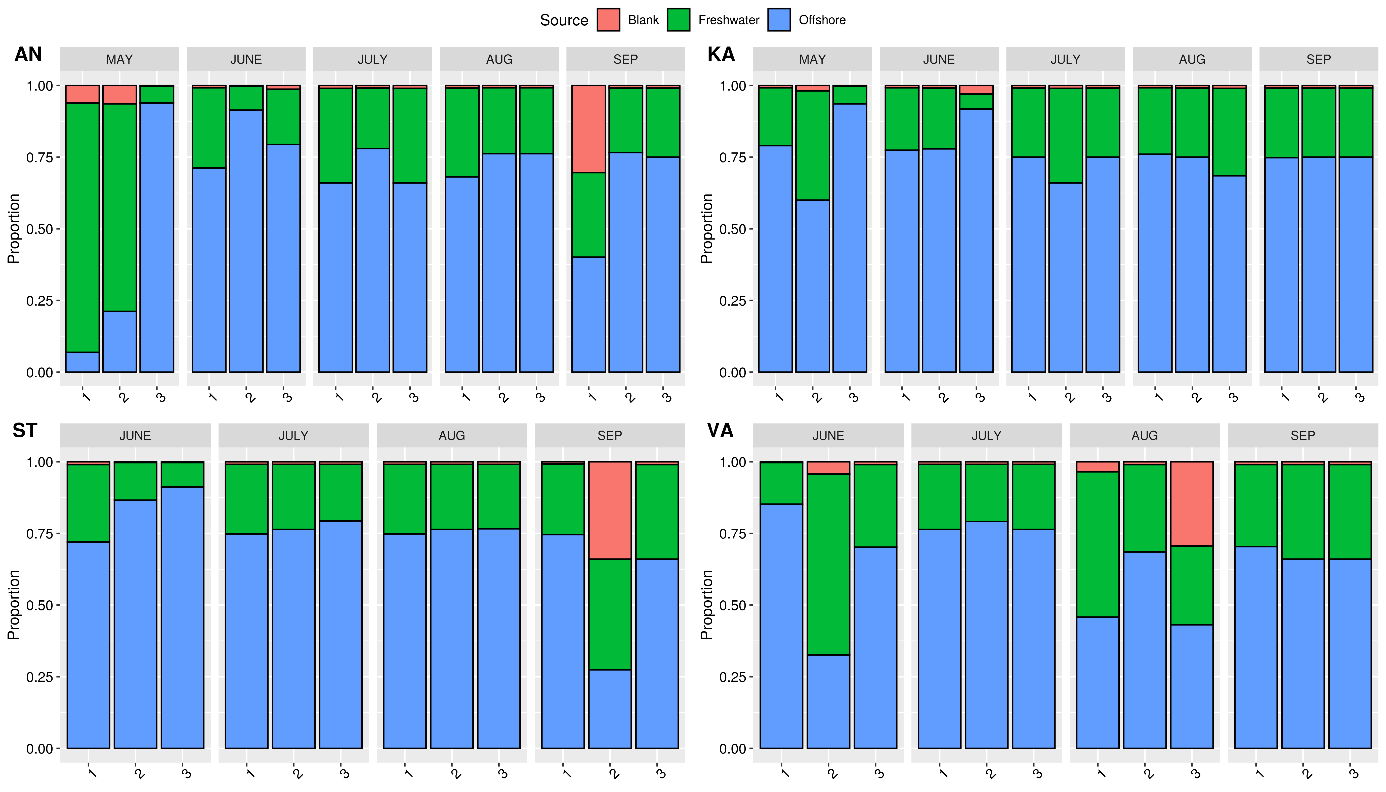


Figure S8: Random Forest analysis of the source prediction for Predation resistant bacteria (PRB) ASVs, the proportion of each source is presented for each bay from May to September.


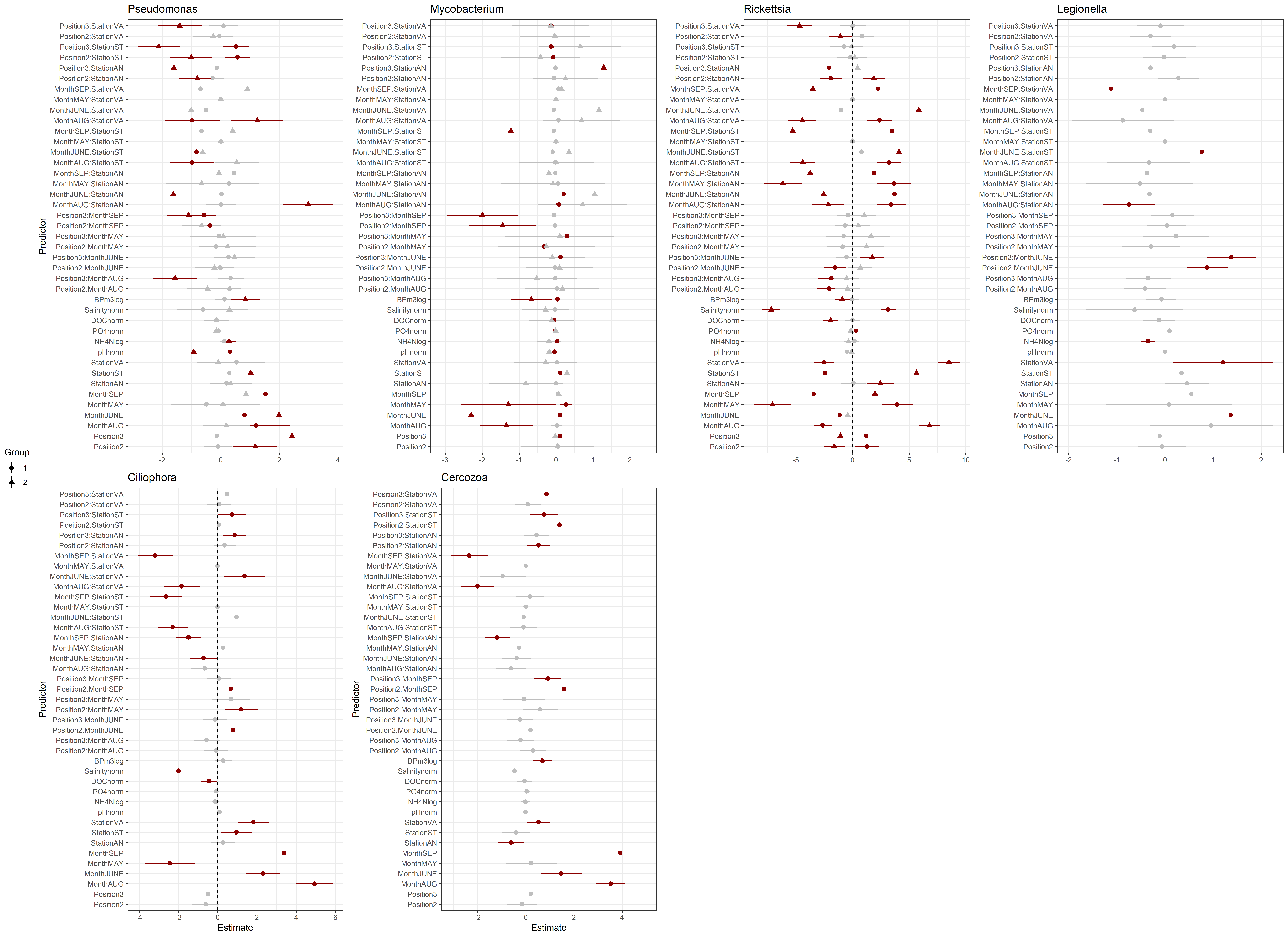


Figure S9: Coefficient estimates by GLLVM analysis showing average effect and uncertainty (as 95% CI) for each Predation resistant bacteria (PRB) and two important protozoa (the rest is not shown here). In the figure July, Kalvarskatan and Position 1 are baselines. Shapes (circle and triangle) are indicating group 1 and 2.


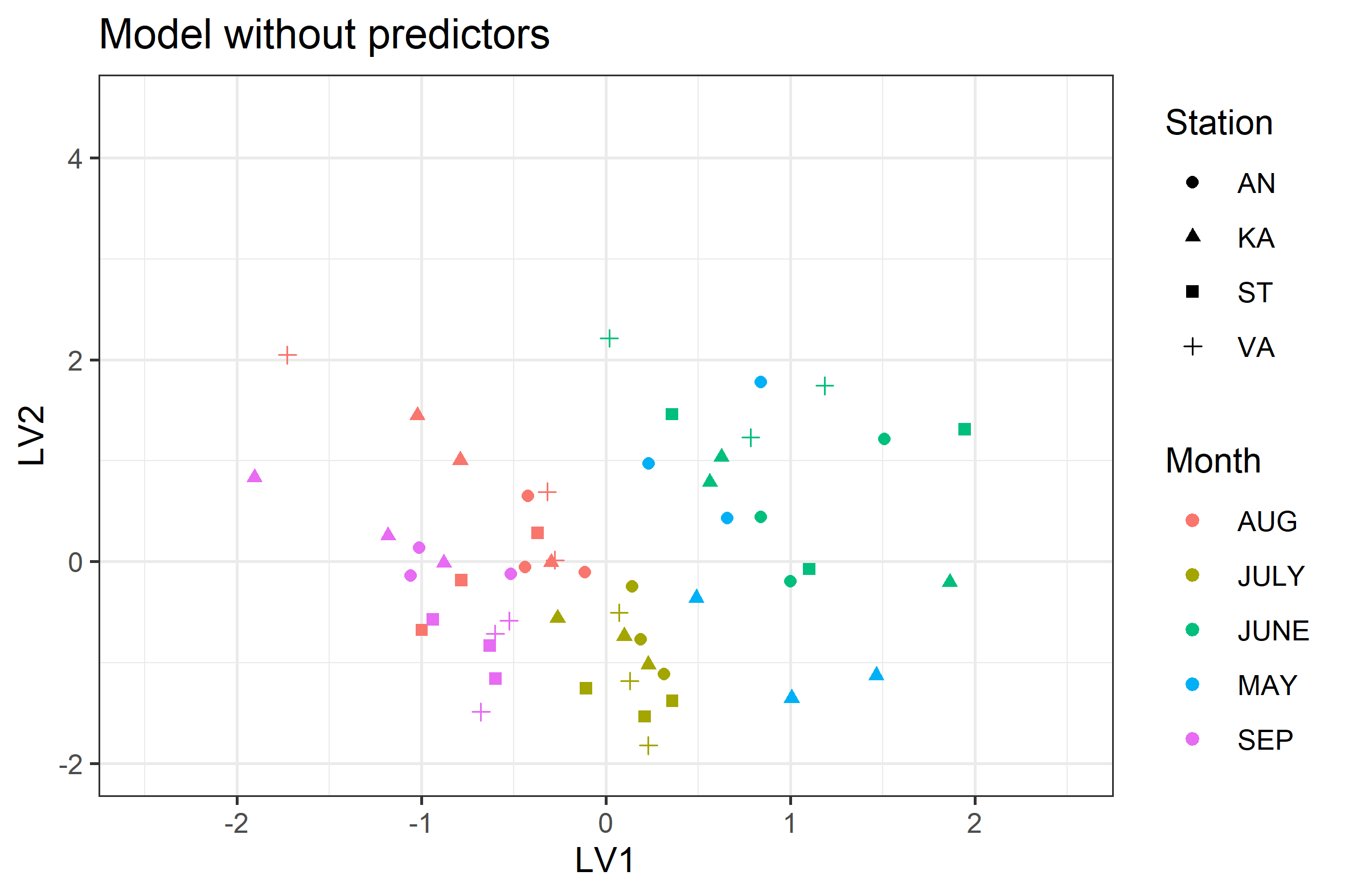


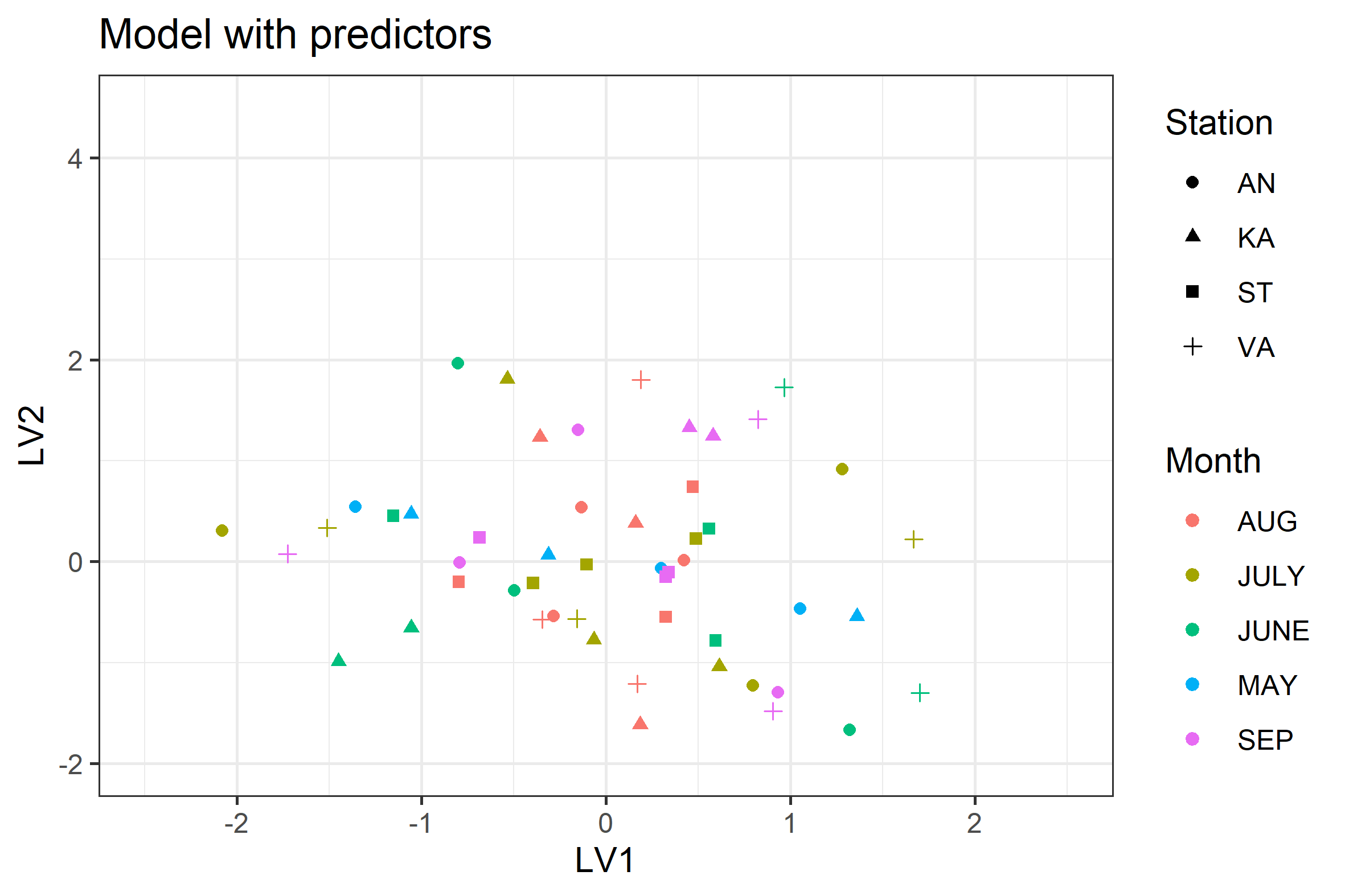


Figure S10: Generalized linear latent variable models (GLLVMs) without and with predictors. The full model with predictors correspond to the following variables: Position*Month + Station*Month + Position*Station + pH + NH_4_ + PO_4_ + DOC + Salinity. The predictors remove the seasonal variation.


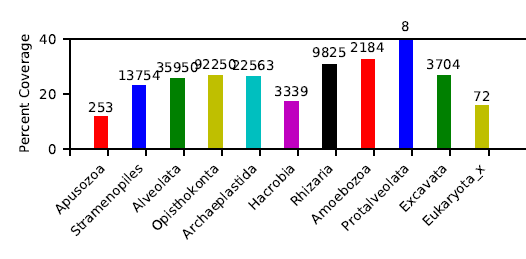


Figure S11: Predicted Taxonomic Coverage of 18S primer pair V6F and V8R, using Primer Prospector (Walters et al. 2011). Sequences in category Eukaryota. Numeric values above bins represent total sequence counts for each set.

Table S1: Fisher's exact test for count data (presence/absence of PRB genus). Comparison between bays, freshwater (FW) and offshore water (OFF). The null hypothesis is that there is no difference between bays, FW and OFF.

| Fisher's Exact Test for Count Data Bacteria | FW vs offshore, p-value | 95 percent confidence interval (2.5%) | 95 percent confidence interval (97.5%) | Bay vs offshore, p-value | 95 percent confidence interval (2.5%) | 95 percent confidence interval (97.5%) |
| --- | --- | --- | --- | --- | --- | --- |
| *Legionella* | 7.72E-04 | 0.006 | 0.394 | 1.66E-05 | 0.003 | 0.210 |
| *Pseudomonas* | 0.189 | 0.062 | 1.511 | 0.189 | 0.007 | 0.244 |
| *Rickettsia* | 0.619 | 0.031 | 3.915 | 0.619 | 0.031 | 3.915 |
| *Mycobacterium* | 0.396 | 0.147 | 1.849 | 0.154 | 0.099 | 1.346 |

Table S2: Influence score in the Direct Acyclic Graph (DAG) inferred network (by BANJO). Representing dependencies between taxa and/or environmental variables (e.g. taxa A leads to taxa B).

| Parent (”taxa A”) | Child  (”taxa B”) | Influence score | Label |
| --- | --- | --- | --- |
| -20 | -16 | 0,6182 | 0 [label="Mycobacterium_Group1"]; |
| -4 | -8 | 0,5839 | 1 [label="Mycobacterium_Group2"]; |
| -13 | -12 | 0,5727 | 2 [label="Rickettsia_Group1"]; |
| -13 | -9 | 0,555 | 3 [label="Rickettsia_Group2"]; |
| -22 | -20 | 0,489 | 4 [label="Legionella_Group1"]; |
| -14 | -19 | 0,4789 | 5 [label="Pseudomonas_Group1"]; |
| -18 | -20 | 0,3923 | 6 [label="Pseudomonas_Group2"]; |
| -22 | -23 | 0,3764 | 7 [label="Ciliophora"]; |
| -13 | -1 | 0,351 | 8 [label="Dinoflagellata"]; |
| -14 | -18 | 0,3273 | 9 [label="Cercozoa"]; |
| -15 | -22 | 0,3273 | 10 [label="Ochrophyta"]; |
| -7 | -4 | 0,3178 | 11 [label="Apicomplexa"]; |
| -8 | 0 | 0,2623 | 12 [label="Telonemia"]; |
| -17 | -23 | 0,2246 | 13 [label="Cryptophyta"]; |
| -12 | -9 | 0,2147 | 14 [label="TDP"]; |
| -8 | -3 | 0,192 | 15 [label="Chl"]; |
| -7 | -2 | 0,1855 | 16 [label="Bacterial_production"]; |
| -21 | -2 | 0,0641 | 17 [label="NH4N"]; |
| -7 | -5 | 0,0321 | 18 [label="pH"]; |
| -13 | -3 | -0,1822 | 19 [label="Salinity"]; |
| -17 | -1 | -0,1964 | 20 [label="DOC"]; |
| -23 | -9 | -0,2273 | 21 [label="SiO2Si"]; |
| -19 | 0 | -0,2623 | 22 [label="PO4"]; |
| -9 | -5 | -0,2944 | 23 [label="NO3N"]; |
| -10 | -2 | -0,3056 |  |
| -18 | -6 | -0,3273 |  |
| -21 | -20 | -0,3512 |  |
| -22 | -19 | -0,4171 |  |
| -20 | -13 | -0,5455 |  |
| -9 | -4 | -0,5801 |  |
| -19 | -10 | -0,6182 |  |

Table S3: List of 18S ASVs included in the Generalized linear latent variable models (GLLVMs) and Direct Acyclic Graph (DAG) analysis.

| Kingdom | Super-group | Division | Class | Order | Family | Genus | Species |
| --- | --- | --- | --- | --- | --- | --- | --- |
| Eukaryota | Alveolata | Ciliophora | Ciliophora_X | Ciliophora_XX | Ciliophora_XXX | Mesodinium | Mesodinium_rubrum |
| Eukaryota | Alveolata | Ciliophora | Spirotrichea | Strombidiida | Strombidiidae_R | Strombidium_R | Strombidium_basimorphum |
| Eukaryota | Alveolata | Ciliophora | Ciliophora_X | Ciliophora_XX | Ciliophora_XXX | Mesodinium | Mesodinium_rubrum |
| Eukaryota | Alveolata | Dinoflagellata | Dinophyceae | Dinophyceae_X | Tovelliaceae | Woloszynskia | Woloszynskia_halophila |
| Eukaryota | Alveolata | Dinoflagellata | Dinophyceae | Peridiniales | Thoracosphaeraceae | NA | NA |
| Eukaryota | Alveolata | Ciliophora | Ciliophora_X | Ciliophora_XX | Ciliophora_XXX | Mesodinium | Mesodinium_rubrum |
| Eukaryota | Alveolata | Ciliophora | Ciliophora_X | Ciliophora_XX | Ciliophora_XXX | Mesodinium | Mesodinium_pulex |
| Eukaryota | Alveolata | Ciliophora | Ciliophora_X | Ciliophora_XX | Ciliophora_XXX | Mesodinium | Mesodinium_pulex |
| Eukaryota | Alveolata | Ciliophora | Ciliophora_X | Ciliophora_XX | Ciliophora_XXX | Mesodinium | Mesodinium_rubrum |
| Eukaryota | Rhizaria | Cercozoa | Filosa-Thecofilosea | Cryomonadida | Rhogostoma-lineage | NA | NA |
| Eukaryota | Rhizaria | Cercozoa | Filosa-Thecofilosea | Ebriida | Ebriidae | Ebria | Ebria_tripartita |
| Eukaryota | Alveolata | Ciliophora | Spirotrichea | Hypotrichia | Halteriidae | NA | NA |
| Eukaryota | Alveolata | Ciliophora | Spirotrichea | Strombidiida | Strombidiidae_R | Strombidiidae_R_X | Strombidiidae_R_X_sp. |
| Eukaryota | Stramenopiles | Ochrophyta | Chrysophyceae | Chrysophyceae_X | Chrysophyceae_Clade-C | Uroglena | Uroglena_americana |
| Eukaryota | Stramenopiles | Ochrophyta | Chrysophyceae | Chrysophyceae_X | Chrysophyceae_Clade-E | Chrysophyceae_Clade-E_X | Chrysophyceae_Clade-E_X_sp. |
| Eukaryota | Alveolata | Apicomplexa | Gregarinomorphea | Eugregarinorida | Gregarinidae | Leidyana2 | NA |
| Eukaryota | Alveolata | Ciliophora | Spirotrichea | Choreotrichida | Strobilidiidae_I | Pelagostrobilidium | Pelagostrobilidium_neptuni |
| Eukaryota | Alveolata | Dinoflagellata | Dinophyceae | Gymnodiniales | Chytriodiniaceae | Chytriodinium | Chytriodinium_roseum |
| Eukaryota | Hacrobia | Telonemia | Telonemia_X | Telonemia_XX | Telonemia-Group-1 | Telonema-Group-1 | Telonema_subtile |
| Eukaryota | Alveolata | Ciliophora | Heterotrichea | Heterotrichea_X | Condylostomatidae | Linostomella | Linostomella_sp. |
| Eukaryota | Stramenopiles | Ochrophyta | Dictyochophyceae | Dictyochophyceae_X | Pedinellales | Pseudopedinella | NA |
| Eukaryota | Alveolata | Ciliophora | Prostomatea_3 | Prostomatea_3_X | Holophryidae | Prorodon | Prorodon_teres |
| Eukaryota | Alveolata | Ciliophora | Spirotrichea | Strombidiida_B | Strombidiida_B_X | Strombidiida_B_XX | Strombidiida_B_XX_sp. |
| Eukaryota | Alveolata | Ciliophora | CONThreeP | CONThreeP_X | Urotrichidae | Urotricha | Urotricha_sp. |
| Eukaryota | Rhizaria | Cercozoa | Filosa-Thecofilosea | Cryomonadida | Protaspa-lineage | Protaspa | NA |
| Eukaryota | Hacrobia | Cryptophyta | Cryptophyceae | Cryptomonadales | Cryptomonadales_X | Cryptomonas | Cryptomonas_obovata |
| Eukaryota | Alveolata | Ciliophora | Spirotrichea | Choreotrichida | Strobilidiidae_G | Strobilidiidae_G_X | Strobilidiidae_G_X_sp. |
| Eukaryota | Hacrobia | Cryptophyta | Cryptophyceae | Cryptomonadales | Cryptomonadales_X | Urgorri | Urgorri_complanatus |
| Eukaryota | Alveolata | Ciliophora | Phyllopharyngea | Cyrtophoria_7 | Hartmannulidae | Trochilioides | Trochilioides_recta |

Table S4: List of ASVs assigned to predation resistant bacteria (PRB).

| ASV | Genus.Venn.diagram | ASV.Offshore | ASV.Bay | ASV.Freshwater | Genus.BANJO.and.GLLVMs | Group | RecodedName |
| --- | --- | --- | --- | --- | --- | --- | --- |
| 00b9f99f3e0628b44c84d13a7c810a51 | Legionella | 0 | 0.000919173968993198 | 0.000919173968993198 | NA | NA | ASV_1 |
| 1ebefc531f7a1d6b09c8ac43de0586c7 | Legionella | 0 | 0.00126178024139161 | 0.00126178024139161 | NA | NA | ASV_2 |
| 1fa08d8e5f217bc3e0819fb84635dabd | Legionella | 0 | 0.00142165573870948 | 0.00191851431032122 | NA | NA | ASV_3 |
| 29c75405489dac1956954a56569d5a62 | Legionella | 0 | 0 | 0.000385102052043792 | NA | NA | ASV_4 |
| 3cac2f62e5cdc722c2742b89448ef219 | Legionella | 0 | 9.01750215190392e-05 | 0 | NA | NA | ASV_5 |
| 42cd2d4ea74820c833d60c93b54ff45e | Legionella | 0 | 0 | 0.000168290806513656 | NA | NA | ASV_6 |
| 5546125333287c44ae1727f3d5b96eb6 | Legionella | 0.00571428702242324 | 0.0121522779238616 | 0 | Legionella | 1 | ASV_7 |
| 59df79ea90f96926fdc0e706a9393d8a | Legionella | 0 | 0.000576015687235738 | 0.000576015687235738 | NA | NA | ASV_8 |
| 5a9e19507efd6cf558f25bb52e6fd829 | Legionella | 0 | 0.00027846184637608 | 0.00027846184637608 | NA | NA | ASV_9 |
| 5ccdc3c4f52d65912ffd3afb34b4fe26 | Legionella | 0 | 0.000817913499154625 | 0 | NA | NA | ASV_10 |
| 6b08b339298c086bfac594e0044a1107 | Legionella | 0.000375982868258872 | 0.00328608850847925 | 0 | Legionella | 1 | ASV_11 |
| 79cf69cc994547ea30142af615a9a7cc | Legionella | 0 | 0.0005269930755561 | 0.0005269930755561 | NA | NA | ASV_12 |
| 8129dfa45ca24d02d1a5a36329578bfc | Legionella | 0 | 0.000269759913676828 | 0.000269759913676828 | NA | NA | ASV_13 |
| 8708117b7004454ea4d3ada13fbcf730 | Legionella | 0 | 0 | 0.000272470829593538 | NA | NA | ASV_14 |
| 91a5bcefb69690331512d2f6fa4cfd40 | Legionella | 0 | 0 | 0.000416720092319528 | NA | NA | ASV_15 |
| b1c8ea58b86e36199a0bc067808ad2da | Legionella | 0 | 0.000220601752558368 | 0.000220601752558368 | NA | NA | ASV_16 |
| b3b3552819de1815145194d1de319df1 | Legionella | 0 | 0.000293028026037633 | 0 | NA | NA | ASV_17 |
| c2141c04b7c94f9c8a8b80bb1a59415e | Legionella | 0 | 1.79534825267731e-05 | 0 | NA | NA | ASV_18 |
| c3c4d1e063c40171245bfeef5f7b7e1e | Legionella | 0 | 0.000748586004214262 | 0.0012574708587007 | NA | NA | ASV_19 |
| c8f112273fb2eb3fd306f1f9a5fe1f75 | Legionella | 0 | 0.000399342868089458 | 0 | NA | NA | ASV_20 |
| 02264bf641a860b7f595cad44536c00c | Mycobacterium | 0 | 0.000582321932082874 | 0 | NA | NA | ASV_21 |
| 06ddfcd0e2c4d034034b7ef6e4d62e66 | Mycobacterium | 0 | 0 | 0.000544069640914037 | NA | NA | ASV_22 |
| 1809cafad72747db4357ccb62252a1ab | Mycobacterium | 0 | 0 | 0.0010179501751054 | NA | NA | ASV_23 |
| 1ab00f6d5c276854565f999600a5f108 | Mycobacterium | 0.0142832956986349 | 0.0946498395177666 | 0.00144469634569972 | Mycobacterium | 1 | ASV_24 |
| 1abb53dffc389ae3218acce6376fcc34 | Mycobacterium | 0 | 0.00258073640404495 | 0.00521158670380953 | Mycobacterium | 2 | ASV_25 |
| 1c0a41b245b48aa722208b4c004c6596 | Mycobacterium | 0.00869853554716561 | 0.061775959998607 | 0.000828743269980569 | Mycobacterium | 1 | ASV_26 |
| 5d5868c29b49d1b193f621384950ae8d | Mycobacterium | 0 | 0 | 0.000817412488780613 | NA | NA | ASV_27 |
| 5e0710dd98730767b70d3c53bbc6b283 | Mycobacterium | 0 | 0 | 0.00052090011539941 | NA | NA | ASV_28 |
| 6ef0ba7972a3d850ab53b4cff1ecc30a | Mycobacterium | 0 | 0 | 0 | NA | NA | ASV_29 |
| 74a26e97b02fcab82b0d346de4920f75 | Mycobacterium | 0 | 0.000785408521114448 | 0.00934422331265307 | Mycobacterium | 2 | ASV_30 |
| 7879c1b3853f3cd41f0f667a8cba469b | Mycobacterium | 0.000112314593175485 | 0 | 0 | NA | NA | ASV_31 |
| 7b9e4dd5e9a9e31c981691b0a2265217 | Mycobacterium | 0 | 0.000102612046654277 | 0 | NA | NA | ASV_32 |
| 883db5d084c1d946916056f5e3a64bd6 | Mycobacterium | 0.00389563342844316 | 0.0262735681739909 | 0 | Mycobacterium | 1 | ASV_33 |
| 99e5a1f89bd0985c7232e7288b0bbb0c | Mycobacterium | 0.000603049229212723 | 0.00184991763643597 | 0.000428930551257201 | Mycobacterium | 2 | ASV_34 |
| 9a66017e03fdedda6651d86eb37a299f | Mycobacterium | 0.00750337150577847 | 0.0513870814386086 | 0.000971790243424618 | Mycobacterium | 1 | ASV_35 |
| a8157578b3f11139476f964081f8c19e | Mycobacterium | 0 | 0.000313685231128982 | 0.00141959624536158 | NA | NA | ASV_36 |
| ad8664f21c744d16b06f829014b5511c | Mycobacterium | 0.000343288705801579 | 0.00665581066515672 | 0 | Mycobacterium | 1 | ASV_37 |
| b0554c9f11b0f29e5cd5b5a2cb857d94 | Mycobacterium | 0 | 0.00306159881979166 | 0.00163375597752421 | Mycobacterium | 2 | ASV_38 |
| bca92bacb095b7b7038fa9db51eecb3a | Mycobacterium | 0 | 0 | 0 | NA | NA | ASV_39 |
| be04e5a5f333faeeb89b7922cc154ccb | Mycobacterium | 0.000857974605666382 | 0.00158069811553527 | 0 | Mycobacterium | 2 | ASV_40 |
| ca527f2b2b745a618c4c486f18a1de58 | Mycobacterium | 0.111291654919872 | 0.700040843894559 | 0.0153240884522772 | Mycobacterium | 1 | ASV_41 |
| d360035e5ff54580b954bc852b4f86a9 | Mycobacterium | 0 | 0.00591681312243702 | 0 | NA | NA | ASV_42 |
| dc833156df5cf571bb4d9ffca16a6fd9 | Mycobacterium | 0.000105294931102017 | 6.84080311028515e-05 | 0.000272470829593538 | NA | NA | ASV_43 |
| ea50ab99242e77fd8d897c2c43449f84 | Mycobacterium | 7.01966207346778e-05 | 0 | 0 | NA | NA | ASV_44 |
| ebc6606308b7fd8d43bd000d55e885d8 | Mycobacterium | 0 | 0.00296186020964508 | 0.0122298858851108 | Mycobacterium | 2 | ASV_45 |
| 0b36c352632cb1346cdc7d73f9fb376c | Pseudomonas | 0 | 0.00643634654025367 | 0.000962755130109479 | Pseudomonas | 1 | ASV_46 |
| 0bacd86c12479e2ab092ac930571a3de | Pseudomonas | 0 | 0 | 0 | NA | NA | ASV_47 |
| 0ddcd311e02f742e2e0e61ce02cf9c29 | Pseudomonas | 0 | 0.000199443903469151 | 0.000455887037204245 | NA | NA | ASV_48 |
| 0f18144d308ada95632ab5193d92073f | Pseudomonas | 0.00583011929321015 | 0.0199078116230299 | 0 | Pseudomonas | 2 | ASV_49 |
| 1bc599cac5647c5c30496e93ca551557 | Pseudomonas | 0 | 0.00298009193714715 | 0.000280484677522759 | NA | NA | ASV_50 |
| 38c27ceaed634984c1225a82648cf571 | Pseudomonas | 0 | 0.00226222522874954 | 0.00127006084288806 | Pseudomonas | 1 | ASV_51 |
| 3ef3bf00702397006fafc0fab1ba9d93 | Pseudomonas | 0 | 0.0075278265963679 | 0 | NA | NA | ASV_52 |
| 41ec397c2f60b378115b3c89b4aac01c | Pseudomonas | 0 | 0.00413005272407733 | 0 | NA | NA | ASV_53 |
| 43fddf1528d4a98928fd8c3a8ac23bfd | Pseudomonas | 0 | 0.000670048817842443 | 0.000670048817842443 | NA | NA | ASV_54 |
| 5648dccee530d68ceb3e4d7d22cf8756 | Pseudomonas | 0.000832776424203336 | 0.0247259575660074 | 0.0137122333076105 | Pseudomonas | 1 | ASV_55 |
| 6376ea6dab7d0fde3cd66f53b57e1484 | Pseudomonas | 0 | 0.00282230751862723 | 0 | NA | NA | ASV_56 |
| 637b9b3f4d1cbb1a10c07817619cdf69 | Pseudomonas | 0 | 0.00110952473955139 | 0.000821128745633924 | NA | NA | ASV_57 |
| 6f3f68e5c8e2a11b388ddbbea9fa182d | Pseudomonas | 0 | 0 | 0 | NA | NA | ASV_58 |
| 80937954b595781f3c64d11de85175a7 | Pseudomonas | 0 | 0.000706882988062799 | 0 | NA | NA | ASV_59 |
| 8b5884acc8c736df09c4260b50dc9297 | Pseudomonas | 0.0380303166203247 | 0.00861576895796763 | 0.0075583949233961 | Pseudomonas | 2 | ASV_60 |
| 9af3467db68cf6063627304cecd46a65 | Pseudomonas | 0.000540513979657019 | 0.00109287150167834 | 0 | Pseudomonas | 1 | ASV_61 |
| bde01b4ad70e7d819b5a70b50dee5477 | Pseudomonas | 0 | 0.00118186545643644 | 0 | NA | NA | ASV_62 |
| c06ccd1f7d57566ef669942328b1a946 | Pseudomonas | 0 | 0.0726668259378895 | 0 | NA | NA | ASV_63 |
| c10442f1117bbfe857fcc8b57429324e | Pseudomonas | 0 | 0 | 0.000343841117896243 | NA | NA | ASV_64 |
| c989e7cc43e4e958af35d45f6d2581f6 | Pseudomonas | 0 | 0.00219594275191424 | 0 | NA | NA | ASV_65 |
| d0c11a5adda368b41592de64d7f45c36 | Pseudomonas | 0 | 0.000330011923011412 | 0 | NA | NA | ASV_66 |
| dd98934b64acf3ad9aa95b5036e6e885 | Pseudomonas | 0 | 0.000357720319945054 | 0 | NA | NA | ASV_67 |
| ee31ec59182e1d7c8682d38b65862aea | Pseudomonas | 0 | 0 | 0 | NA | NA | ASV_68 |
| 190b624b7b51b3d3cda62d8785e2d670 | Rickettsia | 0.0027236288845055 | 0.000169997556285128 | 0 | NA | NA | ASV_69 |
| 2c32110719f7d792854a239ac426a5fd | Rickettsia | 0.00161331554052092 | 0.0183128986952335 | 0.000702456468985483 | Rickettsia | 1 | ASV_70 |
| 34db6c13d5a2ef99e223f350bf64f31f | Rickettsia | 0 | 0.000863696480337124 | 0 | NA | NA | ASV_71 |
| 4d541fab880806d72bbc34338b4f8479 | Rickettsia | 0 | 0 | 0.000266483089427283 | NA | NA | ASV_72 |
| 7f22f25c4471b00326092341600a3ac8 | Rickettsia | 0 | 0 | 0.000655104261508738 | NA | NA | ASV_73 |
| bc14c3802baa188d4efc90adccde8092 | Rickettsia | 0 | 0 | 0.000577653078065687 | NA | NA | ASV_74 |
| bcd53bcc70b51bb73378601e6fdba3bc | Rickettsia | 0.00126207167684843 | 0.00281845033488251 | 0 | Rickettsia | 2 | ASV_75 |
| e7d8e41f59b77514045eef6c22f6cf0e | Rickettsia | 0 | 0.000441185419582547 | 0.000441185419582547 | NA | NA | ASV_76 |

Table S5: Coefficient estimates by Generalized linear latent variable models (GLLVMs) analysis showing average effect and uncertainty (as 95% CI) for each PRB and protozoa. In the table July, Kalvarskatan and Position 1 are baselines.

| ASV | Predictor | Estimate | CI2.5% | CI97.5% |
| --- | --- | --- | --- | --- |
| Apicomplexa | Position2 | -0,002 | -0,010 | 0,006 |
| Apicomplexa | Position3 | 0,003 | -0,006 | 0,013 |
| Apicomplexa | MonthAUG | -0,003 | -0,029 | 0,024 |
| Apicomplexa | MonthJUNE | -0,006 | -0,017 | 0,005 |
| Apicomplexa | MonthMAY | -0,006 | -0,031 | 0,019 |
| Apicomplexa | MonthSEP | -0,004 | -0,025 | 0,017 |
| Apicomplexa | StationAN | -0,003 | -0,011 | 0,005 |
| Apicomplexa | StationST | -0,002 | -0,020 | 0,015 |
| Apicomplexa | StationVA | 0,007 | -0,016 | 0,029 |
| Apicomplexa | pHnorm | 0,003 | 0,000 | 0,007 |
| Apicomplexa | NH4Nlog | 0,000 | -0,002 | 0,003 |
| Apicomplexa | PO4norm | 0,000 | -0,001 | 0,002 |
| Apicomplexa | DOCnorm | -0,004 | -0,010 | 0,002 |
| Apicomplexa | Salinitynorm | -0,007 | -0,028 | 0,014 |
| Apicomplexa | BPm3log | -0,002 | -0,008 | 0,003 |
| Apicomplexa | Position2:MonthAUG | 0,003 | -0,004 | 0,009 |
| Apicomplexa | Position3:MonthAUG | 0,003 | -0,004 | 0,010 |
| Apicomplexa | Position2:MonthJUNE | 0,000 | -0,007 | 0,008 |
| Apicomplexa | Position3:MonthJUNE | -0,003 | -0,012 | 0,006 |
| Apicomplexa | Position2:MonthMAY | -0,006 | -0,015 | 0,003 |
| Apicomplexa | Position3:MonthMAY | -0,014 | -0,025 | -0,003 |
| Apicomplexa | Position2:MonthSEP | 0,008 | 0,002 | 0,014 |
| Apicomplexa | Position3:MonthSEP | -0,001 | -0,008 | 0,006 |
| Apicomplexa | MonthAUG:StationAN | 0,006 | -0,003 | 0,015 |
| Apicomplexa | MonthJUNE:StationAN | 0,009 | -0,001 | 0,019 |
| Apicomplexa | MonthMAY:StationAN | 0,015 | -0,007 | 0,037 |
| Apicomplexa | MonthSEP:StationAN | 0,001 | -0,012 | 0,013 |
| Apicomplexa | MonthAUG:StationST | 0,008 | -0,008 | 0,025 |
| Apicomplexa | MonthJUNE:StationST | 0,005 | -0,007 | 0,017 |
| Apicomplexa | MonthMAY:StationST | 0,000 | 0,000 | 0,000 |
| Apicomplexa | MonthSEP:StationST | -0,002 | -0,020 | 0,016 |
| Apicomplexa | MonthAUG:StationVA | -0,002 | -0,023 | 0,019 |
| Apicomplexa | MonthJUNE:StationVA | 0,008 | -0,006 | 0,021 |
| Apicomplexa | MonthMAY:StationVA | 0,000 | 0,000 | 0,000 |
| Apicomplexa | MonthSEP:StationVA | -0,003 | -0,021 | 0,015 |
| Apicomplexa | Position2:StationAN | -0,003 | -0,010 | 0,004 |
| Apicomplexa | Position3:StationAN | -0,006 | -0,013 | 0,001 |
| Apicomplexa | Position2:StationST | -0,003 | -0,010 | 0,004 |
| Apicomplexa | Position3:StationST | 0,000 | -0,008 | 0,007 |
| Apicomplexa | Position2:StationVA | -0,008 | -0,015 | -0,001 |
| Apicomplexa | Position3:StationVA | -0,012 | -0,021 | -0,003 |
| Cercozoa | Position2 | -0,152 | -0,785 | 0,481 |
| Cercozoa | Position3 | 0,214 | -0,504 | 0,932 |
| Cercozoa | MonthAUG | 3,491 | 2,871 | 4,110 |
| Cercozoa | MonthJUNE | 1,470 | 0,607 | 2,333 |
| Cercozoa | MonthMAY | 0,250 | -0,834 | 1,335 |
| Cercozoa | MonthSEP | 3,897 | 2,771 | 5,023 |
| Cercozoa | StationAN | -0,605 | -1,146 | -0,064 |
| Cercozoa | StationST | -0,432 | -1,012 | 0,148 |
| Cercozoa | StationVA | 0,493 | 0,003 | 0,983 |
| Cercozoa | pHnorm | -0,008 | -0,264 | 0,247 |
| Cercozoa | NH4Nlog | -0,014 | -0,190 | 0,162 |
| Cercozoa | PO4norm | 0,038 | -0,078 | 0,154 |
| Cercozoa | DOCnorm | -0,041 | -0,366 | 0,284 |
| Cercozoa | Salinitynorm | -0,432 | -0,920 | 0,056 |
| Cercozoa | BPm3log | 0,690 | 0,275 | 1,106 |
| Cercozoa | Position2:MonthAUG | 0,303 | -0,226 | 0,832 |
| Cercozoa | Position3:MonthAUG | -0,222 | -0,798 | 0,354 |
| Cercozoa | Position2:MonthJUNE | 0,190 | -0,297 | 0,677 |
| Cercozoa | Position3:MonthJUNE | -0,244 | -0,799 | 0,310 |
| Cercozoa | Position2:MonthMAY | 0,602 | -0,144 | 1,348 |
| Cercozoa | Position3:MonthMAY | -0,077 | -0,950 | 0,796 |
| Cercozoa | Position2:MonthSEP | 1,589 | 1,092 | 2,087 |
| Cercozoa | Position3:MonthSEP | 0,908 | 0,350 | 1,467 |
| Cercozoa | MonthAUG:StationAN | -0,605 | -1,233 | 0,024 |
| Cercozoa | MonthJUNE:StationAN | -0,367 | -0,963 | 0,230 |
| Cercozoa | MonthMAY:StationAN | -0,266 | -1,193 | 0,660 |
| Cercozoa | MonthSEP:StationAN | -1,166 | -1,677 | -0,656 |
| Cercozoa | MonthAUG:StationST | -0,072 | -0,628 | 0,483 |
| Cercozoa | MonthJUNE:StationST | -0,092 | -0,980 | 0,796 |
| Cercozoa | MonthMAY:StationST | 0,000 | 0,000 | 0,000 |
| Cercozoa | MonthSEP:StationST | 0,198 | -0,382 | 0,778 |
| Cercozoa | MonthAUG:StationVA | -1,972 | -2,663 | -1,282 |
| Cercozoa | MonthJUNE:StationVA | -0,963 | -1,938 | 0,012 |
| Cercozoa | MonthMAY:StationVA | 0,000 | 0,000 | 0,000 |
| Cercozoa | MonthSEP:StationVA | -2,317 | -3,098 | -1,535 |
| Cercozoa | Position2:StationAN | 0,519 | 0,024 | 1,014 |
| Cercozoa | Position3:StationAN | 0,447 | -0,068 | 0,962 |
| Cercozoa | Position2:StationST | 1,398 | 0,822 | 1,974 |
| Cercozoa | Position3:StationST | 0,754 | 0,152 | 1,356 |
| Cercozoa | Position2:StationVA | 0,088 | -0,467 | 0,643 |
| Cercozoa | Position3:StationVA | 0,874 | 0,274 | 1,473 |
| Ciliophora | Position2 | -0,597 | -1,321 | 0,128 |
| Ciliophora | Position3 | -0,494 | -1,304 | 0,316 |
| Ciliophora | MonthAUG | 4,877 | 3,934 | 5,820 |
| Ciliophora | MonthJUNE | 2,287 | 1,306 | 3,267 |
| Ciliophora | MonthMAY | -2,393 | -3,737 | -1,049 |
| Ciliophora | MonthSEP | 3,326 | 1,994 | 4,658 |
| Ciliophora | StationAN | 0,253 | -0,378 | 0,885 |
| Ciliophora | StationST | 0,920 | 0,144 | 1,697 |
| Ciliophora | StationVA | 1,772 | 0,997 | 2,547 |
| Ciliophora | pHnorm | 0,096 | -0,192 | 0,384 |
| Ciliophora | NH4Nlog | -0,113 | -0,316 | 0,089 |
| Ciliophora | PO4norm | -0,083 | -0,217 | 0,051 |
| Ciliophora | DOCnorm | -0,431 | -0,820 | -0,041 |
| Ciliophora | Salinitynorm | -1,959 | -2,702 | -1,215 |
| Ciliophora | BPm3log | 0,285 | -0,188 | 0,757 |
| Ciliophora | Position2:MonthAUG | -0,090 | -0,700 | 0,520 |
| Ciliophora | Position3:MonthAUG | -0,560 | -1,197 | 0,076 |
| Ciliophora | Position2:MonthJUNE | 0,773 | 0,205 | 1,341 |
| Ciliophora | Position3:MonthJUNE | -0,155 | -0,801 | 0,492 |
| Ciliophora | Position2:MonthMAY | 1,192 | 0,332 | 2,052 |
| Ciliophora | Position3:MonthMAY | 0,678 | -0,325 | 1,680 |
| Ciliophora | Position2:MonthSEP | 0,678 | 0,102 | 1,254 |
| Ciliophora | Position3:MonthSEP | 0,066 | -0,571 | 0,704 |
| Ciliophora | MonthAUG:StationAN | -0,650 | -1,378 | 0,078 |
| Ciliophora | MonthJUNE:StationAN | -0,705 | -1,416 | 0,006 |
| Ciliophora | MonthMAY:StationAN | 0,318 | -0,840 | 1,476 |
| Ciliophora | MonthSEP:StationAN | -1,462 | -2,108 | -0,816 |
| Ciliophora | MonthAUG:StationST | -2,250 | -2,983 | -1,517 |
| Ciliophora | MonthJUNE:StationST | 0,952 | -0,047 | 1,952 |
| Ciliophora | MonthMAY:StationST | 0,000 | 0,000 | 0,000 |
| Ciliophora | MonthSEP:StationST | -2,600 | -3,385 | -1,815 |
| Ciliophora | MonthAUG:StationVA | -1,796 | -2,697 | -0,895 |
| Ciliophora | MonthJUNE:StationVA | 1,355 | 0,270 | 2,440 |
| Ciliophora | MonthMAY:StationVA | 0,000 | 0,000 | 0,000 |
| Ciliophora | MonthSEP:StationVA | -3,133 | -4,097 | -2,169 |
| Ciliophora | Position2:StationAN | 0,347 | -0,227 | 0,922 |
| Ciliophora | Position3:StationAN | 0,867 | 0,269 | 1,464 |
| Ciliophora | Position2:StationST | 0,051 | -0,612 | 0,713 |
| Ciliophora | Position3:StationST | 0,724 | 0,036 | 1,412 |
| Ciliophora | Position2:StationVA | 0,071 | -0,566 | 0,709 |
| Ciliophora | Position3:StationVA | 0,484 | -0,218 | 1,186 |
| Cryptophyta | Position2 | -0,729 | -1,315 | -0,144 |
| Cryptophyta | Position3 | -0,916 | -1,554 | -0,278 |
| Cryptophyta | MonthAUG | -0,907 | -1,954 | 0,140 |
| Cryptophyta | MonthJUNE | -0,459 | -1,244 | 0,327 |
| Cryptophyta | MonthMAY | -0,945 | -2,185 | 0,295 |
| Cryptophyta | MonthSEP | 0,417 | -0,731 | 1,566 |
| Cryptophyta | StationAN | -0,383 | -0,917 | 0,151 |
| Cryptophyta | StationST | -0,163 | -0,941 | 0,614 |
| Cryptophyta | StationVA | -0,997 | -1,874 | -0,120 |
| Cryptophyta | pHnorm | -0,042 | -0,270 | 0,187 |
| Cryptophyta | NH4Nlog | 0,139 | -0,029 | 0,307 |
| Cryptophyta | PO4norm | -0,170 | -0,281 | -0,059 |
| Cryptophyta | DOCnorm | 0,149 | -0,193 | 0,490 |
| Cryptophyta | Salinitynorm | 0,036 | -0,785 | 0,858 |
| Cryptophyta | BPm3log | -0,297 | -0,678 | 0,083 |
| Cryptophyta | Position2:MonthAUG | 0,237 | -0,265 | 0,739 |
| Cryptophyta | Position3:MonthAUG | 0,781 | 0,294 | 1,267 |
| Cryptophyta | Position2:MonthJUNE | -0,010 | -0,492 | 0,471 |
| Cryptophyta | Position3:MonthJUNE | 0,183 | -0,366 | 0,731 |
| Cryptophyta | Position2:MonthMAY | 0,516 | -0,195 | 1,226 |
| Cryptophyta | Position3:MonthMAY | 0,945 | 0,124 | 1,766 |
| Cryptophyta | Position2:MonthSEP | -0,491 | -0,969 | -0,012 |
| Cryptophyta | Position3:MonthSEP | -0,423 | -0,940 | 0,095 |
| Cryptophyta | MonthAUG:StationAN | 0,154 | -0,456 | 0,763 |
| Cryptophyta | MonthJUNE:StationAN | -0,010 | -0,627 | 0,607 |
| Cryptophyta | MonthMAY:StationAN | -0,621 | -1,697 | 0,455 |
| Cryptophyta | MonthSEP:StationAN | -0,077 | -0,690 | 0,535 |
| Cryptophyta | MonthAUG:StationST | -0,116 | -0,849 | 0,617 |
| Cryptophyta | MonthJUNE:StationST | -0,433 | -1,217 | 0,352 |
| Cryptophyta | MonthMAY:StationST | 0,000 | 0,000 | 0,000 |
| Cryptophyta | MonthSEP:StationST | -1,264 | -2,062 | -0,466 |
| Cryptophyta | MonthAUG:StationVA | 1,578 | 0,681 | 2,474 |
| Cryptophyta | MonthJUNE:StationVA | 0,344 | -0,491 | 1,179 |
| Cryptophyta | MonthMAY:StationVA | 0,000 | 0,000 | 0,000 |
| Cryptophyta | MonthSEP:StationVA | -0,098 | -0,982 | 0,786 |
| Cryptophyta | Position2:StationAN | 0,786 | 0,308 | 1,264 |
| Cryptophyta | Position3:StationAN | 0,838 | 0,341 | 1,335 |
| Cryptophyta | Position2:StationST | 0,495 | -0,043 | 1,033 |
| Cryptophyta | Position3:StationST | 0,642 | 0,090 | 1,194 |
| Cryptophyta | Position2:StationVA | 0,839 | 0,319 | 1,358 |
| Cryptophyta | Position3:StationVA | 0,931 | 0,342 | 1,519 |
| Dinoflagellata | Position2 | -0,045 | -0,236 | 0,146 |
| Dinoflagellata | Position3 | -0,160 | -0,374 | 0,053 |
| Dinoflagellata | MonthAUG | 0,773 | 0,161 | 1,384 |
| Dinoflagellata | MonthJUNE | -0,001 | -0,257 | 0,255 |
| Dinoflagellata | MonthMAY | 1,961 | 1,374 | 2,548 |
| Dinoflagellata | MonthSEP | 0,389 | -0,095 | 0,873 |
| Dinoflagellata | StationAN | 0,182 | -0,003 | 0,367 |
| Dinoflagellata | StationST | 0,524 | 0,118 | 0,930 |
| Dinoflagellata | StationVA | 0,703 | 0,187 | 1,219 |
| Dinoflagellata | pHnorm | 0,019 | -0,057 | 0,095 |
| Dinoflagellata | NH4Nlog | 0,044 | -0,008 | 0,096 |
| Dinoflagellata | PO4norm | -0,011 | -0,044 | 0,023 |
| Dinoflagellata | DOCnorm | -0,200 | -0,339 | -0,060 |
| Dinoflagellata | Salinitynorm | -0,722 | -1,206 | -0,239 |
| Dinoflagellata | BPm3log | -0,015 | -0,134 | 0,105 |
| Dinoflagellata | Position2:MonthAUG | 0,051 | -0,102 | 0,205 |
| Dinoflagellata | Position3:MonthAUG | 0,051 | -0,117 | 0,218 |
| Dinoflagellata | Position2:MonthJUNE | 0,260 | 0,095 | 0,425 |
| Dinoflagellata | Position3:MonthJUNE | 0,350 | 0,144 | 0,556 |
| Dinoflagellata | Position2:MonthMAY | -0,237 | -0,458 | -0,016 |
| Dinoflagellata | Position3:MonthMAY | -0,917 | -1,171 | -0,662 |
| Dinoflagellata | Position2:MonthSEP | 0,041 | -0,104 | 0,186 |
| Dinoflagellata | Position3:MonthSEP | 0,057 | -0,108 | 0,222 |
| Dinoflagellata | MonthAUG:StationAN | -0,065 | -0,267 | 0,137 |
| Dinoflagellata | MonthJUNE:StationAN | -0,135 | -0,363 | 0,094 |
| Dinoflagellata | MonthMAY:StationAN | -2,459 | -2,966 | -1,952 |
| Dinoflagellata | MonthSEP:StationAN | -0,325 | -0,612 | -0,037 |
| Dinoflagellata | MonthAUG:StationST | -0,431 | -0,820 | -0,043 |
| Dinoflagellata | MonthJUNE:StationST | 0,492 | 0,220 | 0,763 |
| Dinoflagellata | MonthMAY:StationST | 0,000 | 0,000 | 0,000 |
| Dinoflagellata | MonthSEP:StationST | -0,546 | -0,965 | -0,127 |
| Dinoflagellata | MonthAUG:StationVA | -0,563 | -1,044 | -0,081 |
| Dinoflagellata | MonthJUNE:StationVA | 0,357 | 0,054 | 0,661 |
| Dinoflagellata | MonthMAY:StationVA | 0,000 | 0,000 | 0,000 |
| Dinoflagellata | MonthSEP:StationVA | -0,361 | -0,772 | 0,050 |
| Dinoflagellata | Position2:StationAN | -0,080 | -0,244 | 0,083 |
| Dinoflagellata | Position3:StationAN | 0,137 | -0,028 | 0,301 |
| Dinoflagellata | Position2:StationST | -0,029 | -0,196 | 0,137 |
| Dinoflagellata | Position3:StationST | 0,076 | -0,098 | 0,250 |
| Dinoflagellata | Position2:StationVA | -0,098 | -0,258 | 0,062 |
| Dinoflagellata | Position3:StationVA | -0,151 | -0,363 | 0,061 |
| Legionella_Group1 | Position2 | -0,058 | -0,557 | 0,442 |
| Legionella_Group1 | Position3 | -0,109 | -0,661 | 0,444 |
| Legionella_Group1 | MonthAUG | 0,959 | -0,224 | 2,143 |
| Legionella_Group1 | MonthJUNE | 1,364 | 0,716 | 2,011 |
| Legionella_Group1 | MonthMAY | 0,074 | -1,161 | 1,308 |
| Legionella_Group1 | MonthSEP | 0,544 | -0,519 | 1,608 |
| Legionella_Group1 | StationAN | 0,456 | -0,025 | 0,936 |
| Legionella_Group1 | StationST | 0,344 | -0,484 | 1,172 |
| Legionella_Group1 | StationVA | 1,206 | 0,203 | 2,209 |
| Legionella_Group1 | pHnorm | 0,001 | -0,204 | 0,205 |
| Legionella_Group1 | NH4Nlog | -0,352 | -0,497 | -0,206 |
| Legionella_Group1 | PO4norm | 0,091 | -0,004 | 0,186 |
| Legionella_Group1 | DOCnorm | -0,119 | -0,445 | 0,206 |
| Legionella_Group1 | Salinitynorm | -0,631 | -1,564 | 0,303 |
| Legionella_Group1 | BPm3log | -0,074 | -0,392 | 0,244 |
| Legionella_Group1 | Position2:MonthAUG | -0,414 | -0,846 | 0,018 |
| Legionella_Group1 | Position3:MonthAUG | -0,349 | -0,798 | 0,100 |
| Legionella_Group1 | Position2:MonthJUNE | 0,882 | 0,453 | 1,312 |
| Legionella_Group1 | Position3:MonthJUNE | 1,378 | 0,879 | 1,876 |
| Legionella_Group1 | Position2:MonthMAY | -0,290 | -0,898 | 0,319 |
| Legionella_Group1 | Position3:MonthMAY | 0,234 | -0,464 | 0,933 |
| Legionella_Group1 | Position2:MonthSEP | 0,041 | -0,366 | 0,448 |
| Legionella_Group1 | Position3:MonthSEP | 0,155 | -0,293 | 0,603 |
| Legionella_Group1 | MonthAUG:StationAN | -0,741 | -1,282 | -0,200 |
| Legionella_Group1 | MonthJUNE:StationAN | -0,319 | -0,884 | 0,246 |
| Legionella_Group1 | MonthMAY:StationAN | -0,531 | -1,609 | 0,547 |
| Legionella_Group1 | MonthSEP:StationAN | -0,373 | -0,993 | 0,247 |
| Legionella_Group1 | MonthAUG:StationST | -0,335 | -1,130 | 0,460 |
| Legionella_Group1 | MonthJUNE:StationST | 0,766 | 0,055 | 1,477 |
| Legionella_Group1 | MonthMAY:StationST | 0,000 | 0,000 | 0,000 |
| Legionella_Group1 | MonthSEP:StationST | -0,305 | -1,161 | 0,551 |
| Legionella_Group1 | MonthAUG:StationVA | -0,875 | -1,848 | 0,097 |
| Legionella_Group1 | MonthJUNE:StationVA | -0,469 | -1,215 | 0,277 |
| Legionella_Group1 | MonthMAY:StationVA | 0,000 | 0,000 | 0,000 |
| Legionella_Group1 | MonthSEP:StationVA | -1,118 | -1,991 | -0,244 |
| Legionella_Group1 | Position2:StationAN | 0,280 | -0,145 | 0,704 |
| Legionella_Group1 | Position3:StationAN | -0,301 | -0,737 | 0,135 |
| Legionella_Group1 | Position2:StationST | -0,014 | -0,477 | 0,450 |
| Legionella_Group1 | Position3:StationST | 0,195 | -0,282 | 0,673 |
| Legionella_Group1 | Position2:StationVA | -0,297 | -0,739 | 0,145 |
| Legionella_Group1 | Position3:StationVA | -0,093 | -0,624 | 0,437 |
| Mycobacterium_Group1 | Position2 | 0,060 | -0,004 | 0,125 |
| Mycobacterium_Group1 | Position3 | 0,111 | 0,042 | 0,180 |
| Mycobacterium_Group1 | MonthAUG | 0,022 | -0,136 | 0,180 |
| Mycobacterium_Group1 | MonthJUNE | 0,115 | 0,036 | 0,195 |
| Mycobacterium_Group1 | MonthMAY | 0,265 | 0,104 | 0,426 |
| Mycobacterium_Group1 | MonthSEP | 0,052 | -0,083 | 0,188 |
| Mycobacterium_Group1 | StationAN | 0,005 | -0,059 | 0,068 |
| Mycobacterium_Group1 | StationST | 0,117 | 0,007 | 0,228 |
| Mycobacterium_Group1 | StationVA | 0,027 | -0,108 | 0,162 |
| Mycobacterium_Group1 | pHnorm | -0,040 | -0,066 | -0,014 |
| Mycobacterium_Group1 | NH4Nlog | 0,035 | 0,016 | 0,054 |
| Mycobacterium_Group1 | PO4norm | -0,019 | -0,031 | -0,007 |
| Mycobacterium_Group1 | DOCnorm | -0,050 | -0,093 | -0,007 |
| Mycobacterium_Group1 | Salinitynorm | -0,032 | -0,156 | 0,092 |
| Mycobacterium_Group1 | BPm3log | 0,044 | 0,004 | 0,084 |
| Mycobacterium_Group1 | Position2:MonthAUG | 0,026 | -0,030 | 0,083 |
| Mycobacterium_Group1 | Position3:MonthAUG | -0,024 | -0,079 | 0,030 |
| Mycobacterium_Group1 | Position2:MonthJUNE | -0,022 | -0,078 | 0,034 |
| Mycobacterium_Group1 | Position3:MonthJUNE | 0,123 | 0,059 | 0,188 |
| Mycobacterium_Group1 | Position2:MonthMAY | -0,321 | -0,400 | -0,241 |
| Mycobacterium_Group1 | Position3:MonthMAY | 0,299 | 0,209 | 0,389 |
| Mycobacterium_Group1 | Position2:MonthSEP | -0,025 | -0,078 | 0,028 |
| Mycobacterium_Group1 | Position3:MonthSEP | -0,047 | -0,104 | 0,010 |
| Mycobacterium_Group1 | MonthAUG:StationAN | 0,071 | 0,001 | 0,142 |
| Mycobacterium_Group1 | MonthJUNE:StationAN | 0,208 | 0,133 | 0,283 |
| Mycobacterium_Group1 | MonthMAY:StationAN | 0,064 | -0,077 | 0,206 |
| Mycobacterium_Group1 | MonthSEP:StationAN | -0,021 | -0,103 | 0,061 |
| Mycobacterium_Group1 | MonthAUG:StationST | -0,012 | -0,118 | 0,093 |
| Mycobacterium_Group1 | MonthJUNE:StationST | -0,085 | -0,175 | 0,004 |
| Mycobacterium_Group1 | MonthMAY:StationST | 0,000 | 0,000 | 0,000 |
| Mycobacterium_Group1 | MonthSEP:StationST | -0,057 | -0,172 | 0,057 |
| Mycobacterium_Group1 | MonthAUG:StationVA | 0,066 | -0,063 | 0,194 |
| Mycobacterium_Group1 | MonthJUNE:StationVA | -0,056 | -0,145 | 0,033 |
| Mycobacterium_Group1 | MonthMAY:StationVA | 0,000 | 0,000 | 0,000 |
| Mycobacterium_Group1 | MonthSEP:StationVA | 0,071 | -0,043 | 0,186 |
| Mycobacterium_Group1 | Position2:StationAN | -0,052 | -0,108 | 0,003 |
| Mycobacterium_Group1 | Position3:StationAN | -0,014 | -0,071 | 0,044 |
| Mycobacterium_Group1 | Position2:StationST | -0,075 | -0,136 | -0,014 |
| Mycobacterium_Group1 | Position3:StationST | -0,126 | -0,189 | -0,064 |
| Mycobacterium_Group1 | Position2:StationVA | -0,028 | -0,086 | 0,030 |
| Mycobacterium_Group1 | Position3:StationVA | -0,127 | -0,198 | -0,057 |
| Mycobacterium_Group2 | Position2 | 0,034 | -0,938 | 1,006 |
| Mycobacterium_Group2 | Position3 | -0,026 | -1,107 | 1,056 |
| Mycobacterium_Group2 | MonthAUG | -1,317 | -2,061 | -0,572 |
| Mycobacterium_Group2 | MonthJUNE | -2,301 | -3,129 | -1,474 |
| Mycobacterium_Group2 | MonthMAY | -1,319 | -2,605 | -0,033 |
| Mycobacterium_Group2 | MonthSEP | 0,083 | -0,936 | 1,102 |
| Mycobacterium_Group2 | StationAN | -0,813 | -1,829 | 0,203 |
| Mycobacterium_Group2 | StationST | 0,320 | -0,684 | 1,325 |
| Mycobacterium_Group2 | StationVA | -0,251 | -1,110 | 0,608 |
| Mycobacterium_Group2 | pHnorm | -0,183 | -0,662 | 0,295 |
| Mycobacterium_Group2 | NH4Nlog | -0,189 | -0,516 | 0,138 |
| Mycobacterium_Group2 | PO4norm | -0,008 | -0,221 | 0,204 |
| Mycobacterium_Group2 | DOCnorm | -0,126 | -0,731 | 0,479 |
| Mycobacterium_Group2 | Salinitynorm | -0,311 | -0,949 | 0,327 |
| Mycobacterium_Group2 | BPm3log | -0,669 | -1,222 | -0,117 |
| Mycobacterium_Group2 | Position2:MonthAUG | 0,169 | -0,831 | 1,169 |
| Mycobacterium_Group2 | Position3:MonthAUG | -0,525 | -1,569 | 0,518 |
| Mycobacterium_Group2 | Position2:MonthJUNE | 0,106 | -0,797 | 1,010 |
| Mycobacterium_Group2 | Position3:MonthJUNE | -0,096 | -0,976 | 0,783 |
| Mycobacterium_Group2 | Position2:MonthMAY | -0,266 | -1,573 | 1,041 |
| Mycobacterium_Group2 | Position3:MonthMAY | 0,099 | -1,370 | 1,568 |
| Mycobacterium_Group2 | Position2:MonthSEP | -1,449 | -2,353 | -0,544 |
| Mycobacterium_Group2 | Position3:MonthSEP | -1,994 | -2,935 | -1,053 |
| Mycobacterium_Group2 | MonthAUG:StationAN | 0,720 | -0,477 | 1,918 |
| Mycobacterium_Group2 | MonthJUNE:StationAN | 1,033 | -0,097 | 2,164 |
| Mycobacterium_Group2 | MonthMAY:StationAN | -0,104 | -1,506 | 1,299 |
| Mycobacterium_Group2 | MonthSEP:StationAN | -0,213 | -1,153 | 0,727 |
| Mycobacterium_Group2 | MonthAUG:StationST | -0,017 | -1,034 | 0,999 |
| Mycobacterium_Group2 | MonthJUNE:StationST | 0,356 | -1,246 | 1,958 |
| Mycobacterium_Group2 | MonthMAY:StationST | 0,000 | 0,000 | 0,000 |
| Mycobacterium_Group2 | MonthSEP:StationST | -1,249 | -2,313 | -0,185 |
| Mycobacterium_Group2 | MonthAUG:StationVA | 0,667 | -0,385 | 1,719 |
| Mycobacterium_Group2 | MonthJUNE:StationVA | 1,172 | -0,085 | 2,428 |
| Mycobacterium_Group2 | MonthMAY:StationVA | 0,000 | 0,000 | 0,000 |
| Mycobacterium_Group2 | MonthSEP:StationVA | 0,132 | -0,884 | 1,148 |
| Mycobacterium_Group2 | Position2:StationAN | 0,260 | -0,624 | 1,144 |
| Mycobacterium_Group2 | Position3:StationAN | 1,298 | 0,365 | 2,232 |
| Mycobacterium_Group2 | Position2:StationST | -0,427 | -1,525 | 0,672 |
| Mycobacterium_Group2 | Position3:StationST | 0,654 | -0,490 | 1,798 |
| Mycobacterium_Group2 | Position2:StationVA | -0,034 | -0,993 | 0,925 |
| Mycobacterium_Group2 | Position3:StationVA | -0,140 | -1,216 | 0,936 |
| Ochrophyta | Position2 | 0,291 | -0,352 | 0,934 |
| Ochrophyta | Position3 | 0,692 | -0,001 | 1,385 |
| Ochrophyta | MonthAUG | 0,456 | -0,511 | 1,424 |
| Ochrophyta | MonthJUNE | 0,187 | -0,599 | 0,972 |
| Ochrophyta | MonthMAY | -2,201 | -3,465 | -0,936 |
| Ochrophyta | MonthSEP | 1,593 | 0,478 | 2,707 |
| Ochrophyta | StationAN | 0,193 | -0,552 | 0,938 |
| Ochrophyta | StationST | -0,119 | -0,959 | 0,722 |
| Ochrophyta | StationVA | 1,011 | 0,147 | 1,874 |
| Ochrophyta | pHnorm | -0,108 | -0,413 | 0,197 |
| Ochrophyta | NH4Nlog | 0,069 | -0,172 | 0,310 |
| Ochrophyta | PO4norm | 0,223 | 0,067 | 0,378 |
| Ochrophyta | DOCnorm | -0,309 | -0,734 | 0,116 |
| Ochrophyta | Salinitynorm | -1,410 | -2,181 | -0,639 |
| Ochrophyta | BPm3log | 0,010 | -0,439 | 0,458 |
| Ochrophyta | Position2:MonthAUG | -0,383 | -1,074 | 0,307 |
| Ochrophyta | Position3:MonthAUG | -0,620 | -1,307 | 0,066 |
| Ochrophyta | Position2:MonthJUNE | -0,050 | -0,721 | 0,620 |
| Ochrophyta | Position3:MonthJUNE | -0,481 | -1,159 | 0,197 |
| Ochrophyta | Position2:MonthMAY | 0,402 | -0,559 | 1,363 |
| Ochrophyta | Position3:MonthMAY | -0,546 | -1,621 | 0,530 |
| Ochrophyta | Position2:MonthSEP | 0,265 | -0,391 | 0,920 |
| Ochrophyta | Position3:MonthSEP | -0,289 | -0,971 | 0,394 |
| Ochrophyta | MonthAUG:StationAN | -0,347 | -1,224 | 0,530 |
| Ochrophyta | MonthJUNE:StationAN | 2,129 | 1,300 | 2,957 |
| Ochrophyta | MonthMAY:StationAN | -1,988 | -3,207 | -0,769 |
| Ochrophyta | MonthSEP:StationAN | -1,414 | -2,174 | -0,655 |
| Ochrophyta | MonthAUG:StationST | 0,922 | 0,099 | 1,746 |
| Ochrophyta | MonthJUNE:StationST | 1,608 | 0,636 | 2,579 |
| Ochrophyta | MonthMAY:StationST | 0,000 | 0,000 | 0,000 |
| Ochrophyta | MonthSEP:StationST | -0,574 | -1,475 | 0,326 |
| Ochrophyta | MonthAUG:StationVA | -0,188 | -1,150 | 0,774 |
| Ochrophyta | MonthJUNE:StationVA | 0,508 | -0,390 | 1,405 |
| Ochrophyta | MonthMAY:StationVA | 0,000 | 0,000 | 0,000 |
| Ochrophyta | MonthSEP:StationVA | 0,159 | -0,759 | 1,077 |
| Ochrophyta | Position2:StationAN | 0,272 | -0,341 | 0,885 |
| Ochrophyta | Position3:StationAN | 0,268 | -0,376 | 0,912 |
| Ochrophyta | Position2:StationST | -0,781 | -1,446 | -0,117 |
| Ochrophyta | Position3:StationST | -0,948 | -1,607 | -0,289 |
| Ochrophyta | Position2:StationVA | -0,825 | -1,480 | -0,170 |
| Ochrophyta | Position3:StationVA | -1,498 | -2,203 | -0,794 |
| Pseudomonas_Group1 | Position2 | -0,099 | -0,581 | 0,383 |
| Pseudomonas_Group1 | Position3 | -0,132 | -0,668 | 0,405 |
| Pseudomonas_Group1 | MonthAUG | 1,228 | -0,060 | 2,516 |
| Pseudomonas_Group1 | MonthJUNE | 0,816 | 0,169 | 1,463 |
| Pseudomonas_Group1 | MonthMAY | -0,504 | -1,803 | 0,794 |
| Pseudomonas_Group1 | MonthSEP | 1,543 | 0,434 | 2,652 |
| Pseudomonas_Group1 | StationAN | 0,204 | -0,253 | 0,660 |
| Pseudomonas_Group1 | StationST | 0,300 | -0,577 | 1,176 |
| Pseudomonas_Group1 | StationVA | 0,550 | -0,535 | 1,636 |
| Pseudomonas_Group1 | pHnorm | 0,317 | 0,124 | 0,510 |
| Pseudomonas_Group1 | NH4Nlog | 0,114 | -0,020 | 0,248 |
| Pseudomonas_Group1 | PO4norm | -0,075 | -0,163 | 0,013 |
| Pseudomonas_Group1 | DOCnorm | -0,147 | -0,470 | 0,177 |
| Pseudomonas_Group1 | Salinitynorm | -0,615 | -1,632 | 0,403 |
| Pseudomonas_Group1 | BPm3log | 0,128 | -0,180 | 0,436 |
| Pseudomonas_Group1 | Position2:MonthAUG | 0,301 | -0,098 | 0,700 |
| Pseudomonas_Group1 | Position3:MonthAUG | 0,338 | -0,086 | 0,762 |
| Pseudomonas_Group1 | Position2:MonthJUNE | -0,005 | -0,413 | 0,404 |
| Pseudomonas_Group1 | Position3:MonthJUNE | 0,267 | -0,225 | 0,759 |
| Pseudomonas_Group1 | Position2:MonthMAY | -0,149 | -0,719 | 0,421 |
| Pseudomonas_Group1 | Position3:MonthMAY | -0,065 | -0,723 | 0,594 |
| Pseudomonas_Group1 | Position2:MonthSEP | -0,377 | -0,755 | 0,001 |
| Pseudomonas_Group1 | Position3:MonthSEP | -0,576 | -1,000 | -0,153 |
| Pseudomonas_Group1 | MonthAUG:StationAN | 0,005 | -0,504 | 0,514 |
| Pseudomonas_Group1 | MonthJUNE:StationAN | 0,020 | -0,527 | 0,566 |
| Pseudomonas_Group1 | MonthMAY:StationAN | 0,249 | -0,876 | 1,373 |
| Pseudomonas_Group1 | MonthSEP:StationAN | 0,439 | -0,200 | 1,077 |
| Pseudomonas_Group1 | MonthAUG:StationST | -1,006 | -1,843 | -0,168 |
| Pseudomonas_Group1 | MonthJUNE:StationST | -0,822 | -1,501 | -0,142 |
| Pseudomonas_Group1 | MonthMAY:StationST | 0,000 | 0,000 | 0,000 |
| Pseudomonas_Group1 | MonthSEP:StationST | -0,678 | -1,581 | 0,225 |
| Pseudomonas_Group1 | MonthAUG:StationVA | -0,993 | -2,028 | 0,042 |
| Pseudomonas_Group1 | MonthJUNE:StationVA | -0,502 | -1,249 | 0,244 |
| Pseudomonas_Group1 | MonthMAY:StationVA | 0,000 | 0,000 | 0,000 |
| Pseudomonas_Group1 | MonthSEP:StationVA | -0,714 | -1,627 | 0,199 |
| Pseudomonas_Group1 | Position2:StationAN | -0,272 | -0,676 | 0,132 |
| Pseudomonas_Group1 | Position3:StationAN | -0,136 | -0,548 | 0,277 |
| Pseudomonas_Group1 | Position2:StationST | 0,567 | 0,136 | 0,998 |
| Pseudomonas_Group1 | Position3:StationST | 0,523 | 0,076 | 0,971 |
| Pseudomonas_Group1 | Position2:StationVA | -0,052 | -0,467 | 0,362 |
| Pseudomonas_Group1 | Position3:StationVA | 0,089 | -0,424 | 0,603 |
| Pseudomonas_Group2 | Position2 | 1,163 | 0,404 | 1,922 |
| Pseudomonas_Group2 | Position3 | 2,427 | 1,585 | 3,270 |
| Pseudomonas_Group2 | MonthAUG | 0,229 | -0,646 | 1,104 |
| Pseudomonas_Group2 | MonthJUNE | 1,985 | 1,015 | 2,954 |
| Pseudomonas_Group2 | MonthMAY | 0,021 | -1,313 | 1,354 |
| Pseudomonas_Group2 | MonthSEP | 0,875 | -0,417 | 2,168 |
| Pseudomonas_Group2 | StationAN | 0,348 | -0,377 | 1,073 |
| Pseudomonas_Group2 | StationST | 1,055 | 0,258 | 1,852 |
| Pseudomonas_Group2 | StationVA | -0,043 | -0,797 | 0,710 |
| Pseudomonas_Group2 | pHnorm | -0,931 | -1,256 | -0,606 |
| Pseudomonas_Group2 | NH4Nlog | 0,273 | 0,038 | 0,508 |
| Pseudomonas_Group2 | PO4norm | -0,153 | -0,307 | 0,000 |
| Pseudomonas_Group2 | DOCnorm | -0,158 | -0,589 | 0,273 |
| Pseudomonas_Group2 | Salinitynorm | 0,251 | -0,444 | 0,945 |
| Pseudomonas_Group2 | BPm3log | 0,831 | 0,335 | 1,327 |
| Pseudomonas_Group2 | Position2:MonthAUG | -0,449 | -1,146 | 0,249 |
| Pseudomonas_Group2 | Position3:MonthAUG | -1,564 | -2,285 | -0,843 |
| Pseudomonas_Group2 | Position2:MonthJUNE | -0,211 | -0,863 | 0,441 |
| Pseudomonas_Group2 | Position3:MonthJUNE | 0,480 | -0,220 | 1,180 |
| Pseudomonas_Group2 | Position2:MonthMAY | 0,236 | -0,734 | 1,206 |
| Pseudomonas_Group2 | Position3:MonthMAY | 0,085 | -1,027 | 1,197 |
| Pseudomonas_Group2 | Position2:MonthSEP | -0,645 | -1,300 | 0,009 |
| Pseudomonas_Group2 | Position3:MonthSEP | -1,095 | -1,803 | -0,388 |
| Pseudomonas_Group2 | MonthAUG:StationAN | 2,969 | 2,123 | 3,815 |
| Pseudomonas_Group2 | MonthJUNE:StationAN | -1,639 | -2,448 | -0,831 |
| Pseudomonas_Group2 | MonthMAY:StationAN | -0,699 | -1,906 | 0,509 |
| Pseudomonas_Group2 | MonthSEP:StationAN | -0,086 | -0,791 | 0,619 |
| Pseudomonas_Group2 | MonthAUG:StationST | 0,508 | -0,264 | 1,279 |
| Pseudomonas_Group2 | MonthJUNE:StationST | -0,613 | -1,712 | 0,485 |
| Pseudomonas_Group2 | MonthMAY:StationST | 0,000 | 0,000 | 0,000 |
| Pseudomonas_Group2 | MonthSEP:StationST | 0,363 | -0,460 | 1,187 |
| Pseudomonas_Group2 | MonthAUG:StationVA | 1,206 | 0,289 | 2,123 |
| Pseudomonas_Group2 | MonthJUNE:StationVA | -1,000 | -2,108 | 0,107 |
| Pseudomonas_Group2 | MonthMAY:StationVA | 0,000 | 0,000 | 0,000 |
| Pseudomonas_Group2 | MonthSEP:StationVA | 0,880 | -0,088 | 1,849 |
| Pseudomonas_Group2 | Position2:StationAN | -0,802 | -1,440 | -0,163 |
| Pseudomonas_Group2 | Position3:StationAN | -1,596 | -2,265 | -0,927 |
| Pseudomonas_Group2 | Position2:StationST | -1,023 | -1,759 | -0,288 |
| Pseudomonas_Group2 | Position3:StationST | -2,125 | -2,880 | -1,371 |
| Pseudomonas_Group2 | Position2:StationVA | -0,265 | -0,967 | 0,437 |
| Pseudomonas_Group2 | Position3:StationVA | -1,416 | -2,182 | -0,650 |
| Rickettsia_Group1 | Position2 | 1,261 | 0,239 | 2,284 |
| Rickettsia_Group1 | Position3 | 1,194 | 0,041 | 2,348 |
| Rickettsia_Group1 | MonthAUG | -2,491 | -3,249 | -1,734 |
| Rickettsia_Group1 | MonthJUNE | -1,107 | -1,911 | -0,304 |
| Rickettsia_Group1 | MonthMAY | 3,817 | 2,514 | 5,120 |
| Rickettsia_Group1 | MonthSEP | -3,344 | -4,334 | -2,354 |
| Rickettsia_Group1 | StationAN | 0,113 | -0,966 | 1,192 |
| Rickettsia_Group1 | StationST | -2,343 | -3,405 | -1,281 |
| Rickettsia_Group1 | StationVA | -2,392 | -3,294 | -1,490 |
| Rickettsia_Group1 | pHnorm | -0,472 | -0,986 | 0,042 |
| Rickettsia_Group1 | NH4Nlog | 0,197 | -0,150 | 0,543 |
| Rickettsia_Group1 | PO4norm | 0,289 | 0,064 | 0,514 |
| Rickettsia_Group1 | DOCnorm | -0,007 | -0,647 | 0,634 |
| Rickettsia_Group1 | Salinitynorm | 3,059 | 2,401 | 3,717 |
| Rickettsia_Group1 | BPm3log | -0,029 | -0,603 | 0,545 |
| Rickettsia_Group1 | Position2:MonthAUG | -2,045 | -3,107 | -0,982 |
| Rickettsia_Group1 | Position3:MonthAUG | -1,883 | -3,017 | -0,749 |
| Rickettsia_Group1 | Position2:MonthJUNE | -1,520 | -2,478 | -0,562 |
| Rickettsia_Group1 | Position3:MonthJUNE | -0,506 | -1,435 | 0,423 |
| Rickettsia_Group1 | Position2:MonthMAY | -0,864 | -2,243 | 0,516 |
| Rickettsia_Group1 | Position3:MonthMAY | -0,760 | -2,312 | 0,792 |
| Rickettsia_Group1 | Position2:MonthSEP | -0,615 | -1,571 | 0,341 |
| Rickettsia_Group1 | Position3:MonthSEP | -0,393 | -1,394 | 0,607 |
| Rickettsia_Group1 | MonthAUG:StationAN | 3,378 | 2,103 | 4,653 |
| Rickettsia_Group1 | MonthJUNE:StationAN | 3,666 | 2,472 | 4,859 |
| Rickettsia_Group1 | MonthMAY:StationAN | 3,568 | 2,109 | 5,027 |
| Rickettsia_Group1 | MonthSEP:StationAN | 1,840 | 0,839 | 2,840 |
| Rickettsia_Group1 | MonthAUG:StationST | 3,147 | 2,061 | 4,232 |
| Rickettsia_Group1 | MonthJUNE:StationST | 0,809 | -0,905 | 2,523 |
| Rickettsia_Group1 | MonthMAY:StationST | 0,000 | 0,000 | 0,000 |
| Rickettsia_Group1 | MonthSEP:StationST | 3,415 | 2,284 | 4,545 |
| Rickettsia_Group1 | MonthAUG:StationVA | 2,289 | 1,173 | 3,404 |
| Rickettsia_Group1 | MonthJUNE:StationVA | -0,976 | -2,296 | 0,344 |
| Rickettsia_Group1 | MonthMAY:StationVA | 0,000 | 0,000 | 0,000 |
| Rickettsia_Group1 | MonthSEP:StationVA | 2,160 | 1,119 | 3,200 |
| Rickettsia_Group1 | Position2:StationAN | -1,886 | -2,820 | -0,952 |
| Rickettsia_Group1 | Position3:StationAN | -2,036 | -3,022 | -1,049 |
| Rickettsia_Group1 | Position2:StationST | -0,218 | -1,385 | 0,950 |
| Rickettsia_Group1 | Position3:StationST | -0,773 | -1,995 | 0,449 |
| Rickettsia_Group1 | Position2:StationVA | 0,836 | -0,175 | 1,847 |
| Rickettsia_Group1 | Position3:StationVA | -0,009 | -1,143 | 1,125 |
| Rickettsia_Group2 | Position2 | -1,655 | -2,585 | -0,725 |
| Rickettsia_Group2 | Position3 | -1,102 | -2,087 | -0,118 |
| Rickettsia_Group2 | MonthAUG | 6,897 | 5,969 | 7,825 |
| Rickettsia_Group2 | MonthJUNE | -0,434 | -1,507 | 0,639 |
| Rickettsia_Group2 | MonthMAY | -7,189 | -8,810 | -5,567 |
| Rickettsia_Group2 | MonthSEP | 2,020 | 0,606 | 3,435 |
| Rickettsia_Group2 | StationAN | 2,463 | 1,273 | 3,653 |
| Rickettsia_Group2 | StationST | 5,719 | 4,624 | 6,814 |
| Rickettsia_Group2 | StationVA | 8,612 | 7,679 | 9,545 |
| Rickettsia_Group2 | pHnorm | -0,078 | -0,553 | 0,397 |
| Rickettsia_Group2 | NH4Nlog | -0,357 | -0,747 | 0,034 |
| Rickettsia_Group2 | PO4norm | -0,153 | -0,403 | 0,098 |
| Rickettsia_Group2 | DOCnorm | -1,965 | -2,601 | -1,329 |
| Rickettsia_Group2 | Salinitynorm | -7,280 | -8,041 | -6,519 |
| Rickettsia_Group2 | BPm3log | -0,916 | -1,595 | -0,236 |
| Rickettsia_Group2 | Position2:MonthAUG | -0,436 | -1,542 | 0,670 |
| Rickettsia_Group2 | Position3:MonthAUG | -0,521 | -1,605 | 0,563 |
| Rickettsia_Group2 | Position2:MonthJUNE | 0,696 | -0,376 | 1,767 |
| Rickettsia_Group2 | Position3:MonthJUNE | 1,765 | 0,736 | 2,794 |
| Rickettsia_Group2 | Position2:MonthMAY | 1,233 | -0,301 | 2,766 |
| Rickettsia_Group2 | Position3:MonthMAY | 1,657 | -0,042 | 3,357 |
| Rickettsia_Group2 | Position2:MonthSEP | 0,498 | -0,550 | 1,546 |
| Rickettsia_Group2 | Position3:MonthSEP | 1,045 | -0,028 | 2,119 |
| Rickettsia_Group2 | MonthAUG:StationAN | -2,173 | -3,591 | -0,755 |
| Rickettsia_Group2 | MonthJUNE:StationAN | -2,579 | -3,876 | -1,283 |
| Rickettsia_Group2 | MonthMAY:StationAN | -6,243 | -7,945 | -4,541 |
| Rickettsia_Group2 | MonthSEP:StationAN | -3,795 | -4,922 | -2,668 |
| Rickettsia_Group2 | MonthAUG:StationST | -4,475 | -5,571 | -3,379 |
| Rickettsia_Group2 | MonthJUNE:StationST | 4,101 | 2,659 | 5,543 |
| Rickettsia_Group2 | MonthMAY:StationST | 0,000 | 0,000 | 0,000 |
| Rickettsia_Group2 | MonthSEP:StationST | -5,372 | -6,594 | -4,151 |
| Rickettsia_Group2 | MonthAUG:StationVA | -4,528 | -5,753 | -3,303 |
| Rickettsia_Group2 | MonthJUNE:StationVA | 5,875 | 4,651 | 7,099 |
| Rickettsia_Group2 | MonthMAY:StationVA | 0,000 | 0,000 | 0,000 |
| Rickettsia_Group2 | MonthSEP:StationVA | -3,547 | -4,747 | -2,347 |
| Rickettsia_Group2 | Position2:StationAN | 1,900 | 0,948 | 2,853 |
| Rickettsia_Group2 | Position3:StationAN | 0,454 | -0,551 | 1,458 |
| Rickettsia_Group2 | Position2:StationST | 0,197 | -0,819 | 1,213 |
| Rickettsia_Group2 | Position3:StationST | -0,071 | -1,057 | 0,916 |
| Rickettsia_Group2 | Position2:StationVA | -1,069 | -2,089 | -0,050 |
| Rickettsia_Group2 | Position3:StationVA | -4,706 | -5,753 | -3,659 |
| Telonemia | Position2 | 0,856 | 0,040 | 1,673 |
| Telonemia | Position3 | 0,584 | -0,324 | 1,493 |
| Telonemia | MonthAUG | -1,790 | -2,557 | -1,022 |
| Telonemia | MonthJUNE | -1,535 | -2,603 | -0,466 |
| Telonemia | MonthMAY | 0,628 | -0,762 | 2,018 |
| Telonemia | MonthSEP | -0,750 | -2,135 | 0,636 |
| Telonemia | StationAN | 0,126 | -0,687 | 0,939 |
| Telonemia | StationST | -0,665 | -1,470 | 0,140 |
| Telonemia | StationVA | -0,167 | -0,829 | 0,495 |
| Telonemia | pHnorm | -0,254 | -0,613 | 0,105 |
| Telonemia | NH4Nlog | 0,248 | -0,018 | 0,513 |
| Telonemia | PO4norm | 0,242 | 0,069 | 0,416 |
| Telonemia | DOCnorm | -0,106 | -0,570 | 0,358 |
| Telonemia | Salinitynorm | 0,857 | 0,243 | 1,470 |
| Telonemia | BPm3log | -0,299 | -0,852 | 0,255 |
| Telonemia | Position2:MonthAUG | -0,073 | -0,852 | 0,706 |
| Telonemia | Position3:MonthAUG | 0,669 | -0,139 | 1,478 |
| Telonemia | Position2:MonthJUNE | -0,346 | -1,079 | 0,386 |
| Telonemia | Position3:MonthJUNE | -0,018 | -0,791 | 0,754 |
| Telonemia | Position2:MonthMAY | -0,471 | -1,559 | 0,616 |
| Telonemia | Position3:MonthMAY | -0,106 | -1,350 | 1,137 |
| Telonemia | Position2:MonthSEP | -1,594 | -2,329 | -0,860 |
| Telonemia | Position3:MonthSEP | -1,595 | -2,386 | -0,803 |
| Telonemia | MonthAUG:StationAN | 0,027 | -0,930 | 0,984 |
| Telonemia | MonthJUNE:StationAN | 1,249 | 0,357 | 2,142 |
| Telonemia | MonthMAY:StationAN | 0,692 | -0,587 | 1,970 |
| Telonemia | MonthSEP:StationAN | 0,928 | 0,165 | 1,691 |
| Telonemia | MonthAUG:StationST | 2,804 | 2,019 | 3,589 |
| Telonemia | MonthJUNE:StationST | 1,267 | 0,076 | 2,459 |
| Telonemia | MonthMAY:StationST | 0,000 | 0,000 | 0,000 |
| Telonemia | MonthSEP:StationST | 2,725 | 1,883 | 3,567 |
| Telonemia | MonthAUG:StationVA | 0,756 | -0,177 | 1,688 |
| Telonemia | MonthJUNE:StationVA | 0,884 | -0,330 | 2,098 |
| Telonemia | MonthMAY:StationVA | 0,000 | 0,000 | 0,000 |
| Telonemia | MonthSEP:StationVA | 5,806 | 4,792 | 6,821 |
| Telonemia | Position2:StationAN | -0,797 | -1,497 | -0,097 |
| Telonemia | Position3:StationAN | -0,754 | -1,488 | -0,020 |
| Telonemia | Position2:StationST | -1,374 | -2,168 | -0,580 |
| Telonemia | Position3:StationST | -0,389 | -1,199 | 0,421 |
| Telonemia | Position2:StationVA | -1,112 | -1,885 | -0,340 |
| Telonemia | Position3:StationVA | -1,849 | -2,671 | -1,027 |

**References**

Andersson, A., S. Brugel, J. Paczkowska, O. F. Rowe, D. Figueroa, S. Kratzer, and C. Legrand. 2018. Influence of allochthonous dissolved organic matter on pelagic basal production in a northerly estuary. Estuarine, Coastal and Shelf Science 204:225–235.

Andersson, A., S. Hajdu, P. Haecky, J. Kuparinen, and J. Wikner. 1996. Succession and growth limitation of phytoplankton in the Gulf of Bothnia (Baltic Sea). Marine Biology 126:791–801.

Gargas, E., C. S. Nielsen, and J. Lønholdt. 1976. An incubator method for estimating the actual daily planktonalgae primary production. Water Research 10:853–860.

Grasshoff, K., K. Kremling, and M. Ehrhardt. 2007. Methods of Seawater Analysis: Third, Completely Revised and Extended Edition. Wiley.

Menden-Deuer, S., and E. J. Lessard. 2000. Carbon to volume relationships for dinoflagellates, diatoms, and other protist plankton. Limnology and Oceanography 45:569–579.

Olenina, I., S. Hajdu, L. Edler, A. Andersson, N. Wasmund, S. Busch, J. Göbel, S. Gromisz, S. Huseby, M. Huttunen, A. Jaanus, P. Kokkonen, and E. Niemkiewicz. 2006. Biovolumes and size-classes of phytoplankton in the Baltic Sea HELCOM. Baltic Sea Environ Proc 106:1–44.

Pullerits, K., S. Chan, J. Ahlinder, A. Keucken, P. Rådström, and C. J. Paul. 2020. Impact of coagulation-ultrafiltration on long-term pipe biofilm dynamics in a full-scale chloraminated drinking water distribution system. Environmental Science: Water Research and Technology 6:3044–3056.

Utermöhl, H. 1958. Zur Vervollkommnung der quantitativen Phytoplankton-Methodik. SIL Communications 9:1–38.

Wikner, J., and Å. Hagström. 1999. Bacterioplankton intra-annual variability: importance of hydrography and competition. Aquatic Microbial Ecology 20:245–260.
